# Longitudinal dynamics of the gut microbiome during methamphetamine addiction and abstinence

**DOI:** 10.3389/fmicb.2026.1911776

**Published:** 2026-09-23

**Authors:** Lingxiao Wang, Jiaqi Wang, Yi Zhang, Baojing Wang, Xiaomeng Sun, Yanhua Li, Jiangwei Yan, Keming Yun, Hongliang Su

**Affiliations:** 1 School of Forensic Medicine, Shanxi Medical University, Taiyuan, China; 2 Technical Appraisal Research Center of the People's Procuratorate of Shanxi Province, Taiyuan, China; 3 Department of Foreign Languages, Shanxi Medical University, Taiyuan, China; 4 Key Laboratory of Forensic Toxicology, Ministry of Public Security, Beijing, China

**Keywords:** abstinence, addiction, gut microbiota, machine learning, methamphetamine

## Abstract

**Introduction:**

Methamphetamine (METH) addiction is a chronic neuropsychiatric disorder characterized by severe abstinence symptoms and high relapse rates, yet the temporal dynamics of gut microbiota alterations across addiction and abstinence phases remain poorly understood.

**Methods:**

In this study, we performed a longitudinal microbiome analysis to characterize differences between phases and time-dependent remodeling of the gut microbiota in a mouse model. Fecal samples were collected at defined time points across a 60-day addiction phase followed by a 60-day abstinence phase and analyzed using 16S rRNA gene sequencing.

**Results:**

Distinct microbial compositions, diversity and community structure differences were observed between addiction and abstinence phases. Microbial communities during the addiction phase exhibited greater dispersion, whereas progressive stabilization was observed during abstinence. Time-series analyses revealed characteristic alterations in *Verrucomicrobia* and *Actinobacteria* during prolonged addiction and early abstinence. At the genus level, *Butyricimonas, Candidatus Arthromitus, Enterococcus*, and *Turicibacter* exhibited reproducible phase- and temporal-dependent dynamic patterns. Based on these microbial signatures, random forest models were constructed for phase classification and temporal prediction. The classification model achieved higher model performance during the addiction phase, whereas performance declined during abstinence, reflected by reduced variance explained and increased heterogeneity in gut microbiota composition. Temporal prediction models further demonstrated differential predictive performance between phases.

**Discussion:**

These findings identify reproducible temporal changes in gut microbiota across addiction and abstinence and highlight candidate microbial signatures for understanding addiction progression and distinguishing different phases.

## 1 Introduction

Methamphetamine (METH) is a potent amphetamine-type stimulant with a high addictive liability and pronounced abstinence symptoms compared with other drugs of abuse (Chan et al., 2019). Chronic METH exposure induces persistent structural and functional alterations in the brain and is strongly associated with a spectrum of neuropsychiatric disorders, including anxiety, depression, and cognitive impairment (Lederer et al., 2016). Beyond its neurotoxic effects, accumulating evidence indicates that METH also exerts substantial toxicity on the gastrointestinal system. Long-term abuse has been linked to myocardial ischemia, mesenteric vasoconstriction, and acute intestinal ischemia, while disruption of intestinal barrier integrity leads to increased gut permeability and systemic inflammatory responses (Kevil et al., 2019; Schwarzbach et al., 2020; Wang et al., 2021).

Emerging evidence suggests that alterations in the gut microbiota may actively contribute to addictive behaviors. In individuals with METH abuse, gut microbiota dysbiosis has been shown to enhance drug reward and impulsivity through immune activation, elevated pro-inflammatory cytokine release, and modulation of neurotransmitter synthesis (Lewin-Epstein et al., 2023). Accordingly, gut microbiota imbalance is increasingly recognized as a hallmark pathological feature of METH addiction, with the microbiota–gut–brain axis playing a central role in regulating both addictive behaviors and abstinence-related responses (Yu et al., 2023).

Advances in microbiome research have provided new perspectives on the biological underpinnings of METH addiction. Experimental studies demonstrate that METH exposure induces pronounced alterations in gut microbial composition and diversity. In animal models, METH addiction is associated with a decreased relative abundance of Firmicutes and a concomitant expansion of *Proteobacteria* and *Desulfobacterota*, along with marked changes in short-chain fatty acid (SCFA)–associated taxa such as *Ruminococcus* and *Prevotella* (Liang et al., 2023). Importantly, fecal microbiota transplantation (FMT) studies further support a causal relationship between gut microbiota dysbiosis and addictive behaviors (Zhang et al., 2023). Conversely, restoration of a healthy microbial community or antibiotic-mediated depletion of gut microbiota has been shown to alleviate METH-induced neurobehavioral abnormalities (Chen et al., 2024), highlighting gut microbiota dysregulation as a critical modulatory factor in METH addiction.

Despite accumulating evidence linking gut microbiota dysbiosis to METH addiction, several critical questions remain unresolved (Ru et al., 2025; Zhang et al., 2025). Most existing studies have primarily relied on cross-sectional designs, focusing on microbiota differences between addicted and control subjects, while largely overlooking the dynamic nature of microbial remodeling during addiction progression and abstinence. In particular, the abstinence phase represents a biologically distinct and clinically relevant period, yet its microbiota-associated features and temporal trajectories remain poorly characterized. Moreover, it is unclear whether gut microbiota alterations induced by METH are transient or persist across different phases and time points following drug cessation. The lack of longitudinal investigations limits our understanding of phase-specific and temporal microbial signatures associated with METH addiction and recovery. Addressing these gaps is essential for elucidating how gut microbiota dynamics are linked to addiction-related phenotypes and abstinence- associated responses.

In this study, we applied microbiome-based approaches to systematically characterize gut microbiota alterations during METH addiction and abstinence using combined cross-sectional and longitudinal analyses. By examining microbial community structures across distinct addiction phases and corresponding time points, we aimed to delineate the dynamic patterns of gut microbiota remodeling associated with METH exposure and abstinence. Furthermore, exploratory classification analyses were performed to evaluate whether microbial features could distinguish different addiction-related states. Collectively, this study provides a microbiota-centered framework for understanding the progression of METH addiction from a gut–brain axis perspective. Our findings also suggest potential translational implications for the interpretation of addiction phases and temporal patterns in METH exposure, while highlighting the dynamic nature of gut microbial responses during addiction and abstinence.

## 2 Materials and methods

### 2.1 Animals and chemicals

Male C57BL/6 mice (7–8 weeks old, body weight 19–22 g) were obtained from SPF (Beijing) Biotechnology Co., Ltd. (Beijing, China). Mice were housed individually under controlled environmental conditions (20–22 °C, 50–70% humidity, 12 h light/dark cycle) with free access to food and water. All mice were allowed to acclimate to the housing conditions for 1 week prior to the start of the experiments. All experimental procedures were approved by the Institutional Animal Care and Use Committee of Shanxi Medical University (No. 2021GLL051, Mar 3, 2021) and performed in accordance with the National Institutes of Health Guide for the Care and Use of Laboratory Animals and relevant Chinese regulations.

*d*-Methamphetamine hydrochloride (National Institutes for Food and Drug Control, NIFDC, Beijing, China) was dissolved in sterile normal saline (0.9% NaCl) prior to administration.

### 2.2 Establishment of the METH addiction and abstinence mouse model

After acclimation period, mice were randomly assigned into methamphetamine-treated group (METH group), saline-treated group (Saline group), and control group (Control group; *n* = 8), to establish mouse models of methamphetamine addiction and abstinence. Methamphetamine was administered at a dose of 1 mg/kg. The experiment comprised a 60-day METH administration period followed by a 60-day abstinence period (Figure 1). The duration of METH exposure was selected based on previous evidence that repeated administration induces stable behavioral sensitization lasting for at least 8 weeks (Kuribara et al., 1996; Itzhak and Ali, 2002). Following METH discontinuation, mice were observed for an additional 60 days to monitor gut microbiota dynamics during prolonged abstinence (Li et al., 2015). METH (1 mg/kg, i.p.) was administered once daily throughout the administration period. The dosing regimen was based on our previous study showing that 1 mg/kg produces reliable conditioned place preference in C57BL/6 mice, whereas doses of 0.25 and 5 mg/kg are ineffective in inducing significant reward-related behavior (Su et al., 2013, 2020).

**Figure 1:**
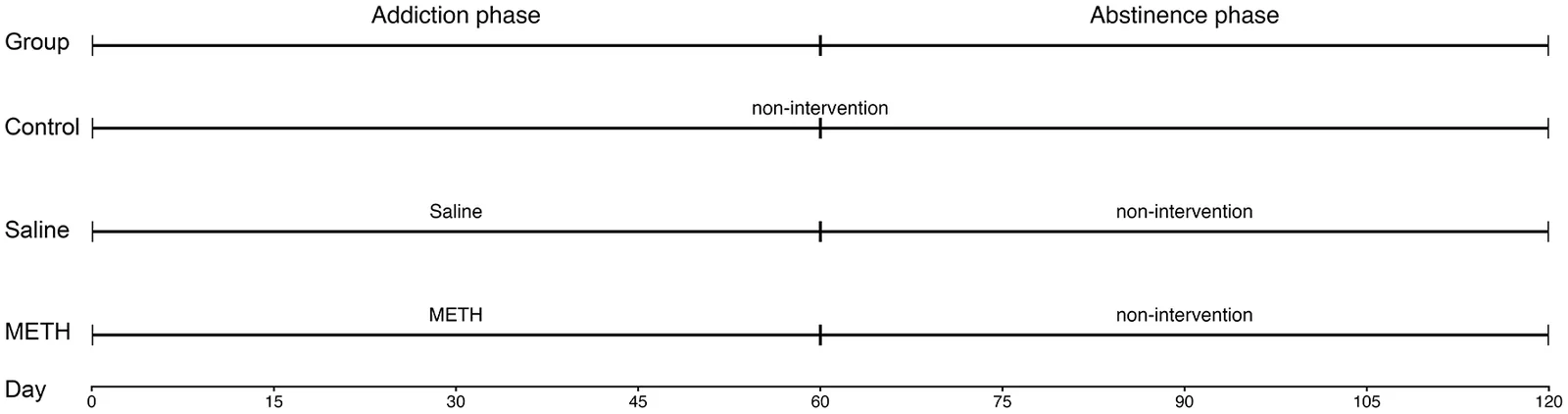
Drug administration flowchart. Control group, control group; Saline group, saline group; METH group, methamphetamine group.

During the addiction phase, mice in the METH and Saline groups received daily intraperitoneal injections of METH or sterile saline, respectively, whereas mice in the Control group received no treatment. No pharmacological intervention was administered during the subsequent abstinence phase. Fecal samples were collected from each mouse at predetermined time points throughout the 120-day experimental period. Approximately 50 mg of fresh feces was collected from each mouse at each sampling time point. Fecal samples were collected separately for each animal rather than from bedding material to maintain the longitudinal correspondence between samples and individual mice. Samples were immediately transferred into sterile 1.5-mL microcentrifuge tubes and stored at −80 °C until DNA extraction. Each experimental group consisted of eight mice, which is comparable to sample sizes used in previous longitudinal murine microbiome studies (Ding et al., 2026).

### 2.3 Gut microbial DNA extraction and 16S rRNA amplicon MiSeq sequencing

Gut microbial DNA was extracted from approximately 50 mg of each fecal sample using the QIAamp PowerFecal Pro DNA Kit (QIAGEN, Germany) according to the manufacturer's instructions. The concentration of total DNA was quantified using a Qubit dsDNA HS Assay Kit on a Qubit 2.0 Fluorometer (Thermo Fisher Scientific, USA).

Sequencing libraries were prepared following the Illumina 16S Metagenomic Sequencing Library Preparation workflow. The V3–V4 hypervariable regions of the bacterial 16S rRNA gene were amplified using universal primers 341F (5′-CCTACGGGNGGCWGCAG-3′) and 806R (5′-GGACTACHVGGGTWTCTAAT-3′). PCR amplification was performed in a total volume of 50 μL, containing 10 μL of 5 × Super Pfx HF Buffer (CoWin Biosciences, China), 6 μL of dNTP Mix (10 mM each), 0.5 μL of Super Pfx DNA Polymerase (CoWin Biosciences), 10 μL of template DNA, and 2 μL of each primer (10 μM).

The PCR cycling conditions were as follows: initial denaturation at 98 °C for 2 min; 26 cycles of denaturation at 98 °C for 10 s, annealing at 55 °C for 30 s, and extension at 72 °C for 20 s; followed by a final extension at 72 °C for 5 min. Amplification was performed using a GeneAmp PCR System 9700 (Applied Biosystems, USA). The first-round PCR products were purified using AMPure XP beads (Beckman Coulter, USA).

For index PCR, 10 μL of purified amplicons were subjected to a second-round PCR in a total volume of 25 μL containing indexed primers, 5 μL of 5 × Super Pfx HF Buffer, 3 μL of dNTP Mix (10 mM each), and 0.25 μL of Super Pfx DNA Polymerase. The PCR conditions were as follows: initial denaturation at 98 °C for 3 min; 12 cycles of denaturation at 98 °C for 10 s, annealing at 55 °C for 30 s, and extension at 72 °C for 8 s; followed by a final extension at 72 °C for 5 min.

The resulting libraries were purified using AMPure XP beads and quantified using the KAPA Library Quantification Kit (Kapa Biosystems, USA) on an Applied Biosystems™ QuantStudio™ 5 Real-Time PCR System (Thermo Fisher Scientific, USA). Purified libraries were diluted to a final concentration of 8 pM, spiked with 10% PhiX control, and sequenced on the Illumina MiSeq FGX platform (Illumina, Inc., USA) using paired-end 2 × 300 bp reads.

### 2.4 Sequencing data processing and statistical analysis

Raw sequencing data were processed using QIIME version 1.9 (Caporaso et al., 2010) and the MicrobiomeAnalyst platform (Dhariwal et al., 2017) for sequence assembly, quality filtering, and clustering, with all parameters set to default unless otherwise specified. Samples with fewer than 1,000 sequences were excluded as low-quality samples. High-quality sequences were clustered into operational taxonomic units (OTUs) at a 97% sequence similarity threshold. Taxonomic assignment of representative sequences was performed using the Greengenes 13_8 reference database (McDonald et al., 2012).

Prior to community-level diversity analyses, sequence counts were rarefied to the minimum sequencing depth across all samples to reduce potential bias arising from differences in sequencing depth. This normalization was used for subsequent α- and β-diversity and community structure analyses. Alpha diversity was assessed by using the Shannon diversity index in Microbiome Analyst. Differences in Shannon diversity across the sampling time points were initially assessed using the non-parametric Kruskal–Wallis test. To characterize temporal changes, exploratory pairwise comparisons between adjacent sampling time points were subsequently performed using Dunn's test, with *P*-values adjusted for multiple comparisons. Beta diversity was evaluated using Bray–Curtis dissimilarity to characterize differences in microbial community composition and relative abundance across experimental groups and time points. Bray–Curtis dissimilarities were calculated in R using the *vegan* package, and community dissimilarities were visualized by principal coordinates analysis (PCoA). A two-sided *P*-value < 0.05 was considered statistically significant.

### 2.5 Biomarker identification and machine learning analysis

Differentially abundant taxa between methamphetamine addiction and abstinence phases were identified using linear discriminant analysis effect size (LEfSe) implemented in Microbiome Analyst, with a Kruskal–Wallis test used for statistical significance and linear discriminant analysis (LDA) used to estimate effect size. Taxa with an LDA score above the predefined threshold were considered potential microbial biomarkers.

Random forest (RF) algorithms were used as exploratory machine-learning approaches for two purposes: classification of addiction vs. abstinence phases and temporal prediction within each phase. All models were constructed using genus-level relative abundance profiles as input features, and all analyses were performed in Microbiome Analyst with default parameters unless otherwise specified.

#### 2.5.1 Phase classification

For classification of METH addiction and abstinence phases, feature importance was first estimated from an initial random forest model using the default importance measure provided by Microbiome Analyst. The top 15 genera with the highest importance scores were selected as candidate features and used to build the final classification model. Model performance was evaluated using out-of-bag (OOB) error and confusion matrices.

#### 2.5.2 Temporal prediction

For temporal prediction analyses, random forest regression models were constructed separately for the addiction and abstinence phases. Genus-level relative abundances were used as input variables, and sampling time points (days) served as the continuous outcome variable. For each phase, the dataset was randomly divided into a training set (70%) and a testing set (30%) at the sample level. Model performance was assessed using the coefficient of determination (*R*^2^), mean absolute error (MAE), and root mean square error (RMSE), calculated separately for the training and testing datasets. Because the analyses were based on a single dataset with longitudinal samples from the same animals and no external validation was available, all machine learning results were interpreted as exploratory.

## 3 Results

### 3.1 Microbiome-based identification of phase-specific gut microbiota signatures and construction of exploratory models during METH addiction and abstinence

After quality filtering of the raw sequencing data (Phred score ≥ Q20), high-quality reads were retained for downstream analyses. A total of 4,306,249 high-quality bacterial reads were obtained from 159 samples, with detailed sequencing information summarized in Supplementary Table S1. To minimize the impact of sequencing depth variability, all samples were rarefied to the minimum read count (4,285 reads per sample). Rarefaction curves demonstrated adequate sequencing depth for downstream microbial community analyses (Supplementary Figure S1). In total, 2,758 operational taxonomic units (OTUs) were identified across all samples. Taxonomic classification against the Greengenes 13_8 reference database assigned these OTUs to 14 bacterial phyla and 99 genera.

#### 3.1.1 Alpha diversity of the gut microbiota during METH addiction and abstinence

Alpha diversity was assessed using the Shannon index. As shown in Figure 2A, no significant differences in Shannon diversity were observed among the experimental groups (*P* > 0.05). Kruskal–Wallis analysis further confirmed the absence of significant differences between the Control and Saline groups (*P* > 0.05). As illustrated in Figure 2B, Shannon index values indicated that microbial richness and evenness during the addiction phase were significantly lower than those observed during the abstinence phase across the three groups (Mann–Whitney *U* = 1,792, *P* < 0.001).

**Figure 2:**
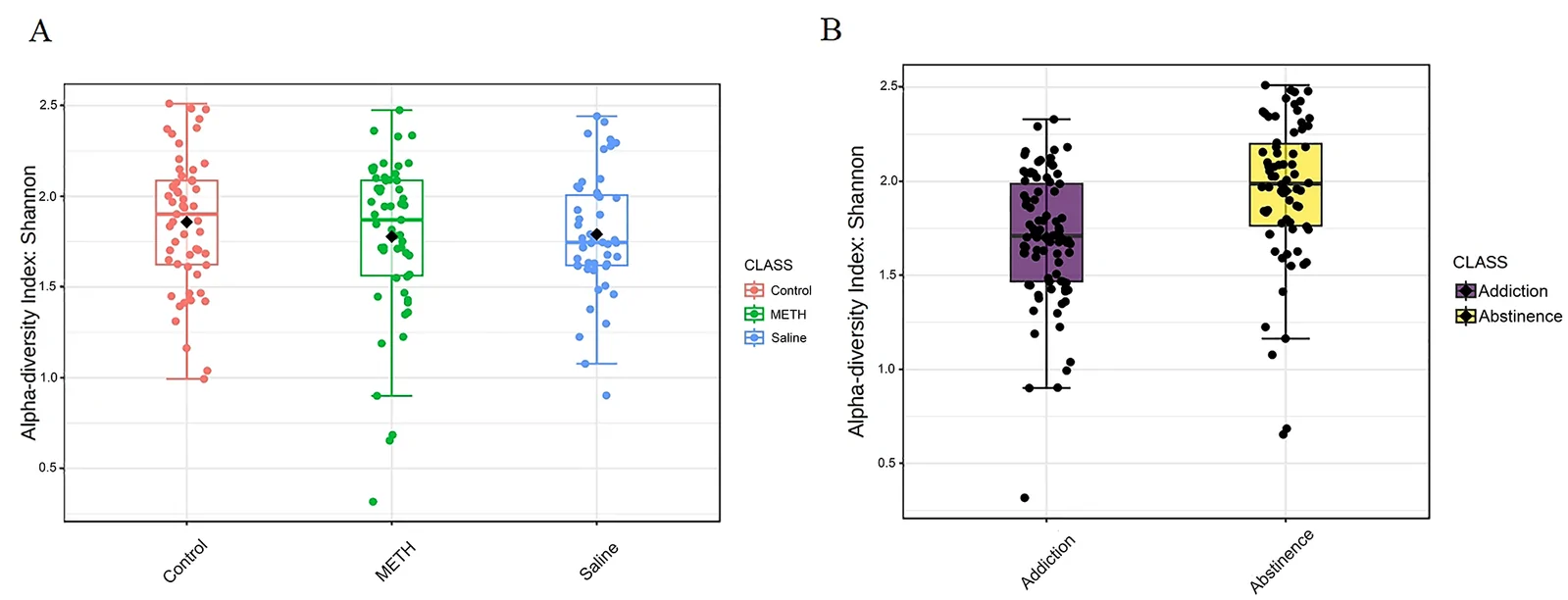
Analysis of gut microbiota alpha diversity using the Shannon index. **(A)** Comparison across experimental groups. No significant difference in the Shannon index was observed among the groups (*P* > 0.05, Kruskal–Wallis test). Specifically, *post-hoc* analysis showed no difference between the control and saline groups (*P* > 0.05). **(B)** Comparison between addiction and abstinence phases. The Shannon index, representing species richness and evenness, was significantly lower during the addiction phase compared to the abstinence phase across all groups (Mann–Whitney *U* = 1792, *P* < 0.001).

#### 3.1.2 Taxonomic composition of the gut microbiota during METH addiction and abstinence

Relative abundance analysis of gut microbial communities at the phylum and genus levels revealed distinct compositional shifts between METH addiction and abstinence phases. At the phylum level (Supplementary Table S2), the gut microbiota of the Control and Saline groups was dominated by *Firmicutes* (36.59%−76.57%) and *Bacteroidetes* (26.15%−60.68%). In contrast, the METH group was primarily characterized by *Firmicutes* (31.22%−76.35%), *Bacteroidetes* (21.25%−54.64%), and *Verrucomicrobia* (0%−31.29%; Figure 3). At the genus level (Supplementary Table S3 and Figure 4), *Enterococcus, Lactobacillus*, and other genera exhibited relatively high abundance and predominance in the Control and Saline groups. During the addiction phase, the gut microbial composition of the METH group differed markedly from that of the Control group, with a reduced relative abundance of *Enterococcus* and an increased abundance of *Bifidobacterium* and *Turicibacter*. During the abstinence phase, partial recovery of the microbial community composition was observed; however, the overall profile remained distinct from both the Control and Saline groups. Linear discriminant analysis effect size (LEfSe) identified *Dehalobacterium* as the discriminative genus at the genus level between the addiction and abstinence phases in the METH group (Supplementary Figure S2).

**Figure 3:**
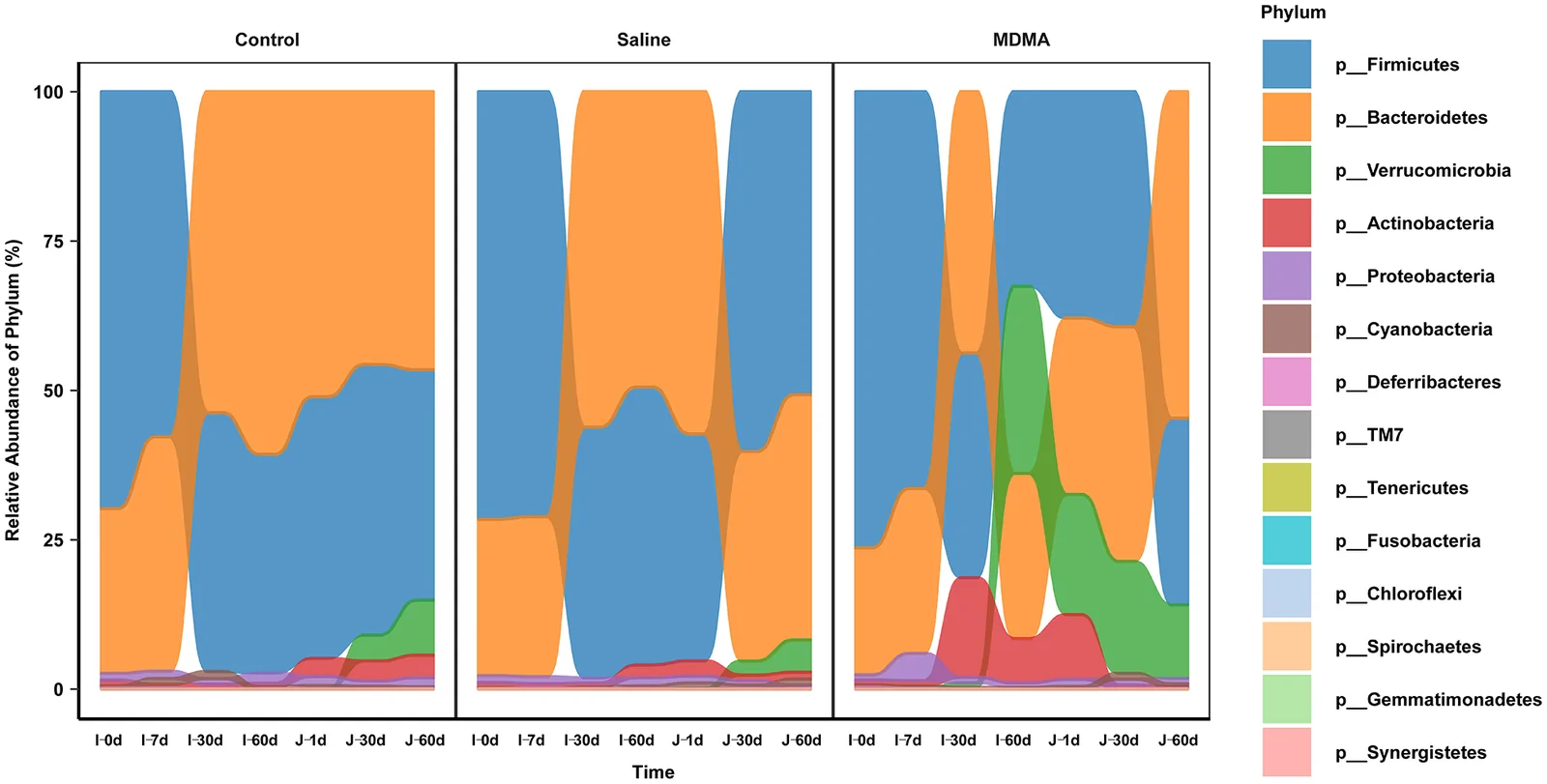
The relative abundance of microbial communities at the phylum level. The *x*-axis represents different time points, and the *y*-axis represents the species abundance at the phylum level; Control: Control group, Saline: Normal saline group, METH: Methamphetamine group.

**Figure 4:**
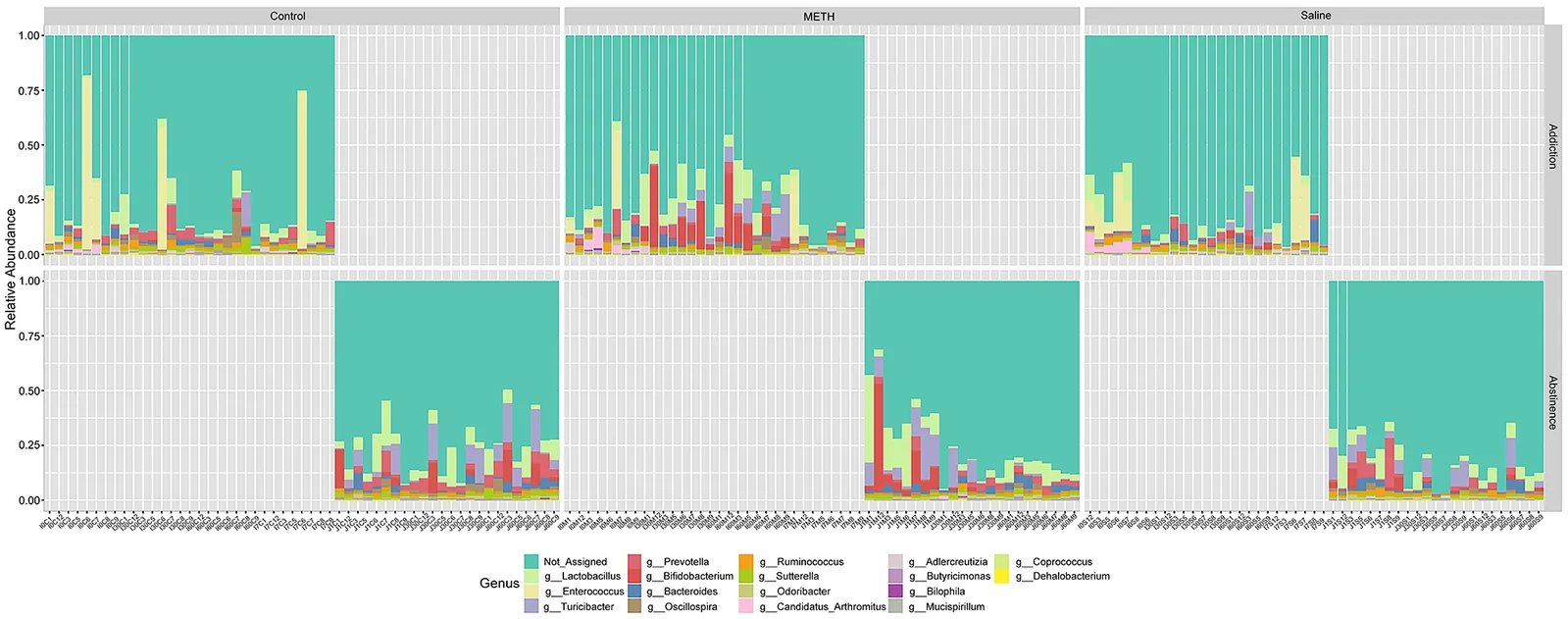
The relative abundance of microbial communities at the genus level. The *x*-axis represents individual experimental mice at different time points; the *y*-axis represents the species abundance at the phylum level. Control, control group; Saline, saline group; METH, methamphetamine group.

#### 3.1.3 Beta diversity of the gut microbiota during METH addiction and abstinence

Beta diversity was assessed using principal coordinates analysis (PCoA) based on Bray–Curtis distances to evaluate differences in gut microbial community structure between addiction and abstinence phases. The PCoA results were visualized in two-dimensional space. In the analysis comparing addiction and abstinence phases within the METH group, the first principal coordinate (PC1) and second principal coordinate (PC2) explained 42.0% and 17.4% of the total variation, respectively, together accounting for the majority of inter-sample variability (Figure 5). The PCoA plot revealed a separation trend in microbial community structure between the addiction and abstinence phases. Samples from the addiction phase were primarily distributed in the upper-left region of the ordination space and exhibited greater dispersion, whereas samples from the abstinence phase clustered predominantly in the lower-left region. Analysis of similarities (ANOSIM) confirmed that the difference between phases was statistically significant (*P* < 0.05).

**Figure 5:**
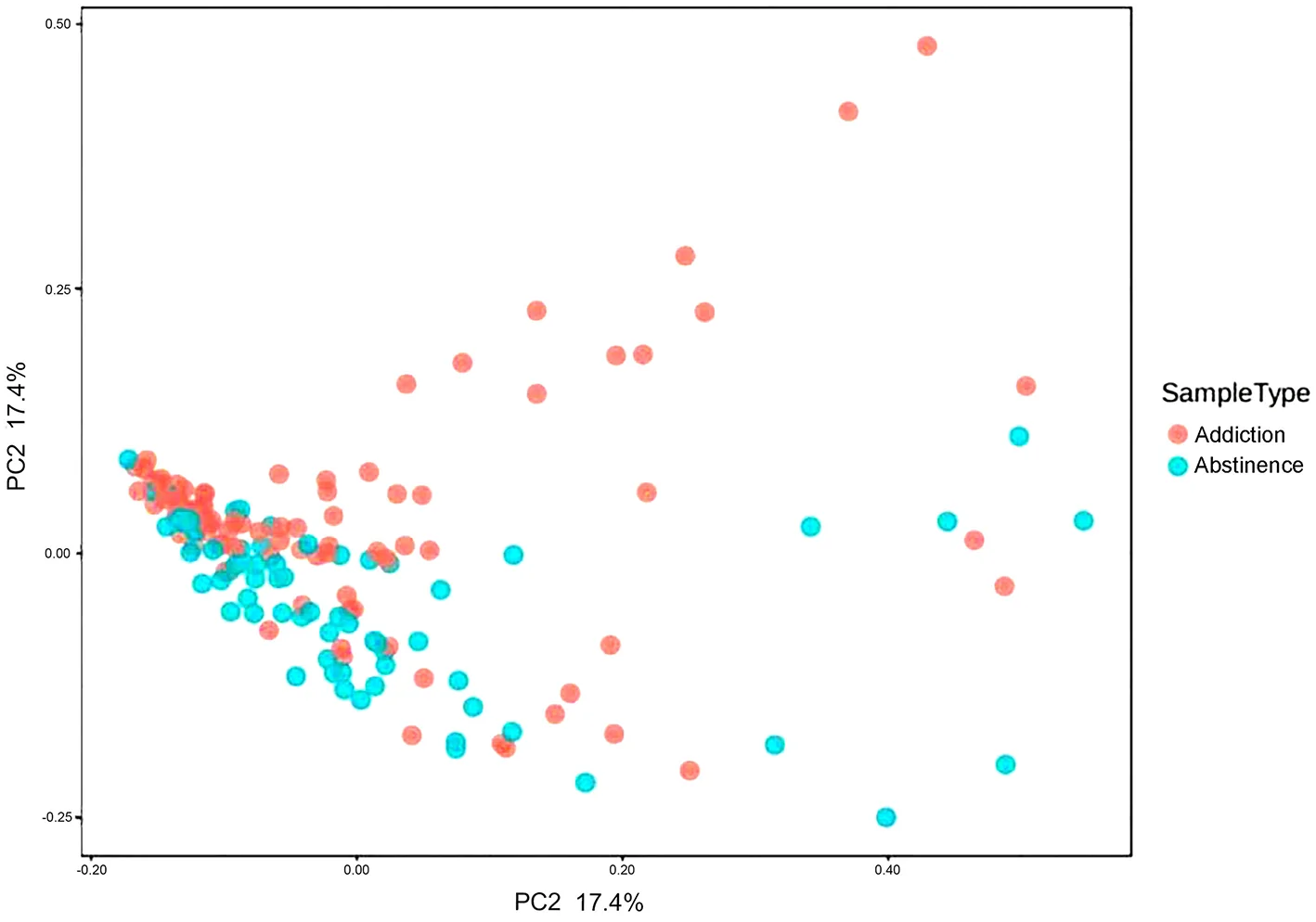
PCoA of gut microbial communities in the METH group between addiction and abstinence phases. Principal Coordinates Analysis (PCoA) was performed based on the Bray–Curtis distance. The first two principal coordinates, PC1 and PC2, explained 42.0% and 17.4% of the total variance, respectively. These two axes captured the majority of the variation among samples.

#### 3.1.4 Exploratory classification of METH use phases based on gut microbiota

To identify gut microbial features associated with discrimination between METH addiction and abstinence phases, genus-level relative abundance profiles were subjected to random forest classification. Feature importance was first estimated using an initial random forest model, and the top 15 genera with the highest importance scores were selected as candidate features for subsequent model construction (Figure 6A). Among these selected features, *Arthrobacter* exhibited the highest importance score, followed by *Butyricimonas*, indicating that these genera contributed substantially to phase discrimination.

**Figure 6:**
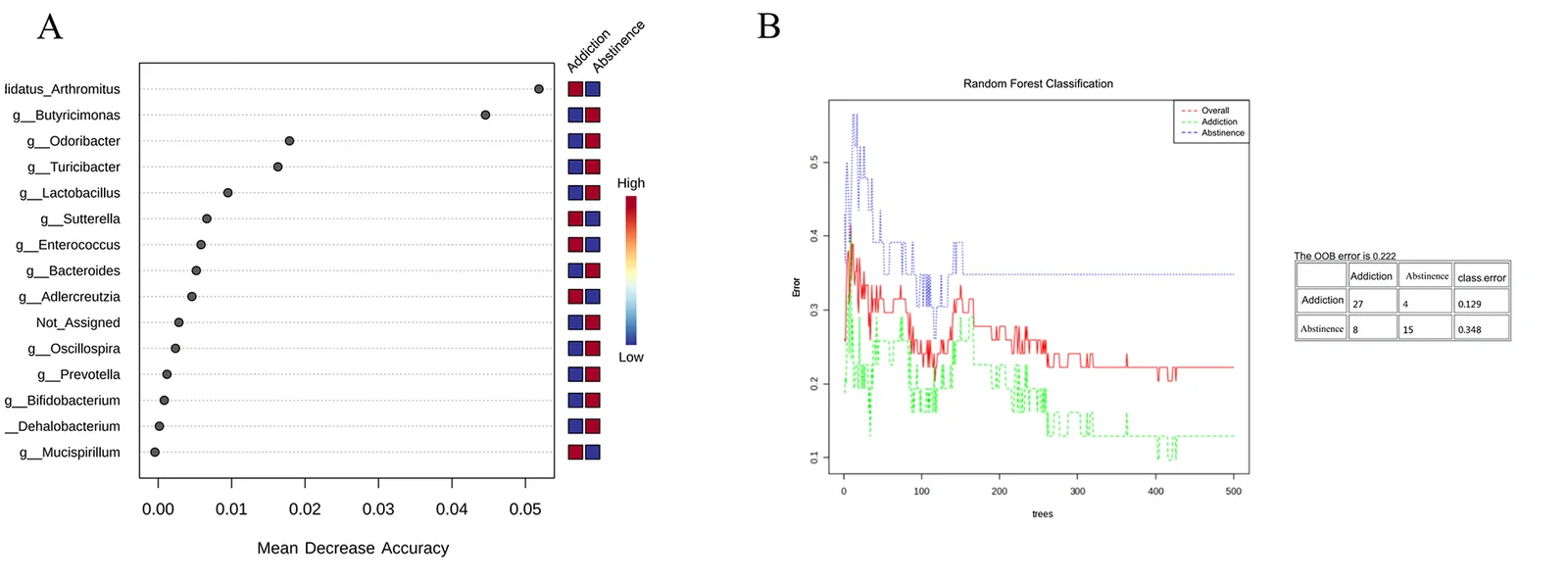
Construction and evaluation of an exploratory random forest classification model for METH use phases based on core microbial genera. **(A)** Fifteen core bacterial genera were selected as biomarkers for the model. *Arthrobacter* was the most important taxon, followed by *Butyricimonas*. **(B)** The out-of-bag (OOB) error of the model stabilized as the number of trees increased. The final model achieved an OOB error of 0.222, with error rates of 0.129 for the addiction phases and 0.348 for the abstinence phases.

Using this candidate feature set, a random forest classifier was constructed to distinguish between addiction and abstinence samples. Model performance was evaluated using the out-of-bag (OOB) error, which provides an internal estimate of model performance based on bootstrap sampling. As shown in Figure 6B, the overall OOB error rate and class-specific error rates decreased progressively with increasing numbers of trees and stabilized after sufficient trees were included. The final confusion matrix revealed classification error rates of 0.129 for the addiction phase and 0.348 for the abstinence phase, with an overall OOB error rate of 0.222. The higher classification error observed for abstinence samples may reflect greater variability in gut microbial composition during abstinence compared with the addiction phase.

Because the classification model was evaluated using internal validation within the current dataset, the results should be interpreted as exploratory.

### 3.2 Microbiome-based identification of temporal gut microbiota signatures during METH addiction and abstinence

#### 3.2.1 Taxonomic composition of the gut microbiota at different time points during METH addiction and abstinence

Stacked bar plots were used to illustrate temporal changes in the relative abundance of gut microbial taxa at the phylum and genus levels across different time points during METH addiction and abstinence (Supplementary Figure S3). At the phylum level (Supplementary Figure S3A), during the addiction phase, *Bacteroidetes* and *Firmicutes* were the dominant phyla from day 0 to day 7. At day 30 of addiction, *Bacteroidetes, Firmicutes*, and *Actinobacteria* became the predominant phyla. By day 60 of addiction, the microbial community was mainly composed of *Verrucomicrobia, Bacteroidetes*, and *Firmicutes*, with a marked increase in the relative abundance of *Verrucomicrobia*. During the abstinence phase, the dominant phyla at abstinence day 1 were similar to those observed at addiction day 60. From abstinence day 30 to day 60, *Bacteroidetes* and *Firmicutes* again became the predominant phyla. Notably, the relative abundance of *Verrucomicrobia* increased from addiction day 60 and gradually decreased after abstinence day 1, whereas *Actinobacteria* increased from addiction day 30 and returned to near-baseline levels after abstinence day 30.

At the genus level (Supplementary Figure S3B), temporal variation in microbial composition was also observed throughout the addiction and abstinence phases. During early addiction (day 0 to day 7), dominant genera included *Lactobacillus, Prevotella, Candidatus Arthromitus*, and *Ruminococcus*. From addiction day 30 to day 60, the dominant genera shifted to *Bifidobacterium, Lactobacillus, Akkermansia*, and *Turicibacter*. During the abstinence phase, *Akkermansia, Lactobacillus, Bifidobacterium*, and *Turicibacter* were predominant at abstinence day 1, whereas from abstinence day 30 to day 60, *Akkermansia, Lactobacillus, Prevotella*, and *Bacteroides*became dominant.

Linear discriminant analysis effect size (LEfSe) identified *Butyricimonas, Candidatus Arthromitus, Enterococcus*, and *Turicibacter* as genera whose abundance patterns were closely associated with different time points during addiction and abstinence. Specifically, the relative abundance of *Butyricimonas* (Figure 7A) differed significantly across time points and showed a gradual increase with prolonged addiction and abstinence. In contrast, *Candidatus Arthromitus* (Figure 7B) decreased progressively with increasing addiction duration and remained at low abundance during abstinence. *Enterococcus* (Figure 7C) exhibited relatively high abundance at addiction day 0 to day 7 but declined markedly by addiction day 60 and throughout the abstinence phase. Conversely, *Turicibacter* (Figure 7D) showed low abundance during early addiction but increased significantly by addiction day 60 and remained elevated during abstinence.

**Figure 7:**
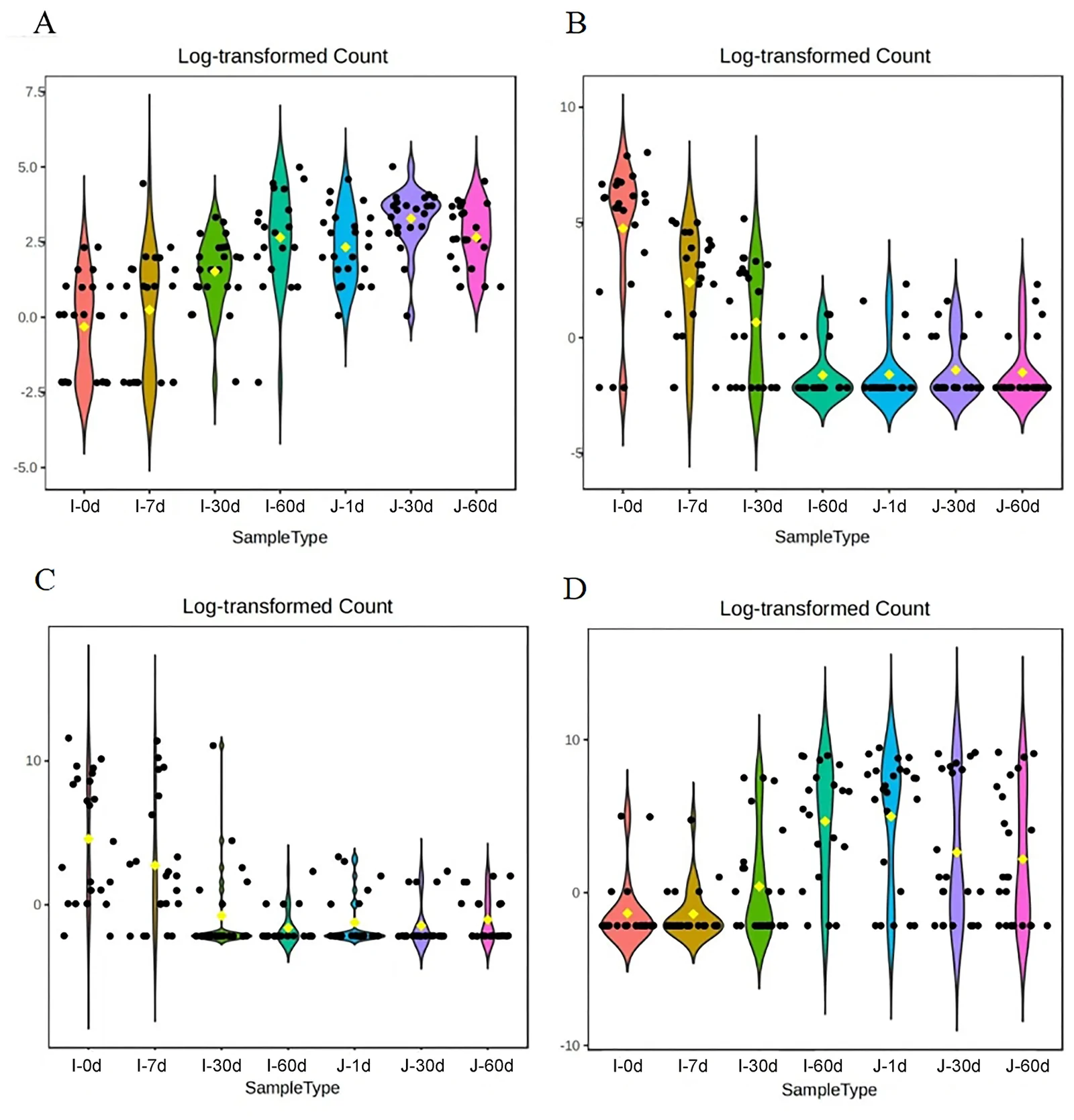
Temporal dynamics of key bacterial genera during METH addiction (I) and abstinence phases (J). LEfSe analysis identified *Butyricimonas, Candidatus Arthromitus, Enterococcus*, and *Turicibacteras* time-associated genera. **(A)** *Butyricimonas* increased with longer addiction/abstinence. **(B)** *Candidatus Arthromitus* decreased during addiction and remained low. **(C)** *Enterococcus* was high early in addiction (day 0–7) but fell by day 60 and during abstinence. **(D)** *Turicibacter* was low in early addiction, rose by day 60, and stayed elevated in abstinence.

#### 3.2.2 Temporal alpha diversity of the gut microbiota during METH addiction and abstinence

Across the seven sampling time points spanning the addiction and abstinence phases, no significant overall difference in Shannon diversity was detected (Kruskal–Wallis *H* = 5.2871, *P* = 0.51). Exploratory pairwise comparisons between adjacent time points nevertheless identified significant differences at several consecutive intervals (Figure 8). During the addiction phase, the Shannon diversity index significantly decreased from addiction day 0 to day 7 (*P* < 0.001), followed by significant increases from day 7 to day 30 and from day 30 to day 60 (*P* < 0.01). No significant difference was observed between addiction day 60 and abstinence day 1. During the abstinence phase, Shannon diversity significantly increased from abstinence day 1 to day 30 (*P* < 0.01), followed by a significant decrease from day 30 to day 60 (*P* < 0.001). These findings indicate transient fluctuations in alpha diversity over the course of METH addiction and abstinence, despite the absence of a significant overall difference across the seven time points.

**Figure 8:**
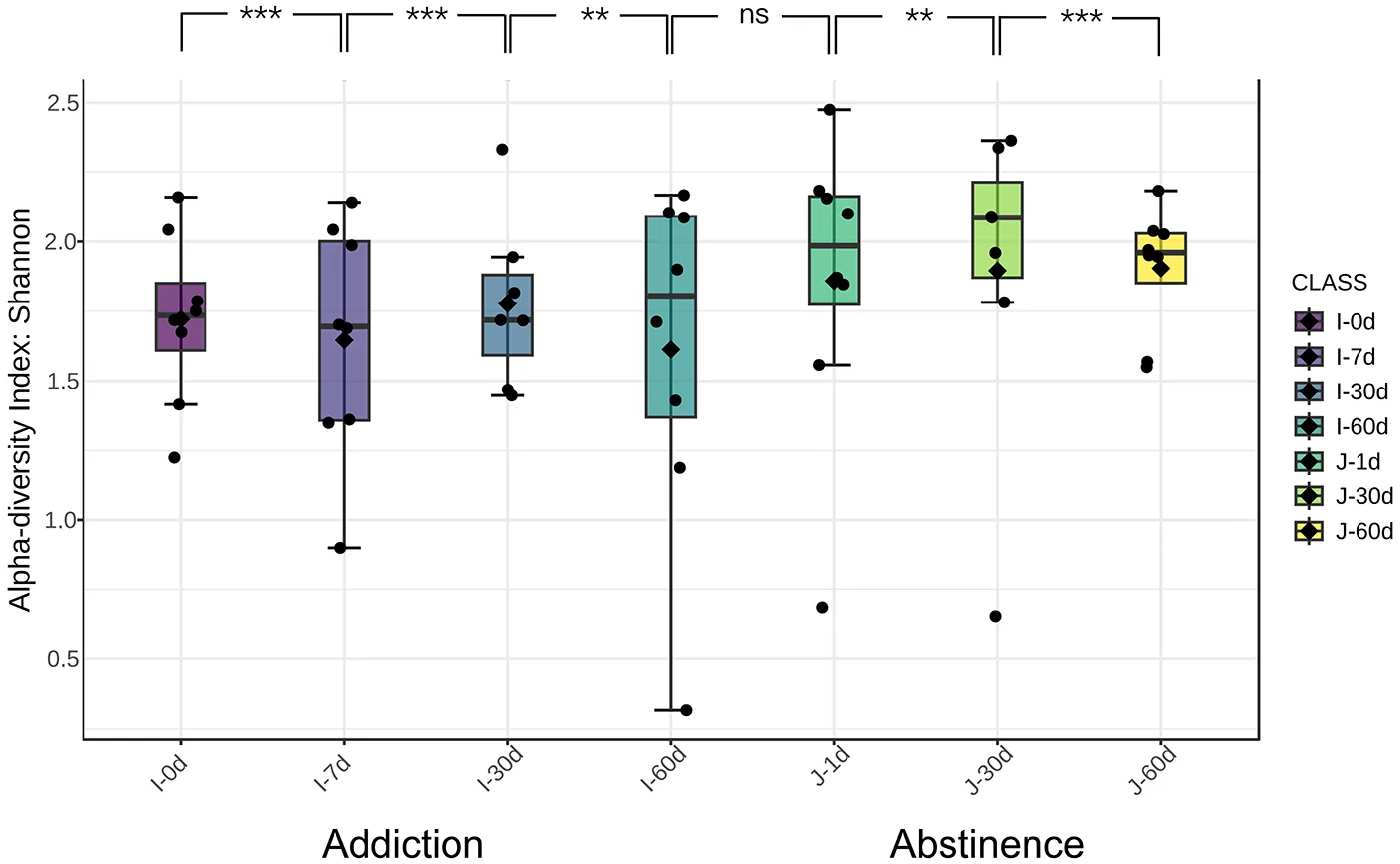
Temporal variation of microbial alpha diversity (Shannon index) during addiction (I) and abstinence (J). Shannon diversity showed no significant overall difference across the seven sampling time points (Kruskal–Wallis *H* = 5.2871, *P* = 0.51). Exploratory pairwise comparisons between adjacent time points revealed a significant decrease from addiction day 0 to day 7, followed by significant increases from day 7 to day 30 and from day 30 to day 60. No significant difference was observed between addiction day 60 and abstinence day 1. During abstinence, Shannon diversity significantly increased from day 1 to day 30 and decreased from day 30 to day 60. ***P < 0.001; **P < 0.01; ns, not significant.

#### 3.2.3 Temporal beta diversity of the gut microbiota during METH addiction and abstinence

Beta diversity across different time points during METH addiction and abstinence was assessed using principal coordinates analysis (PCoA) based on Bray–Curtis distances (Figure 9), enabling visualization of dynamic changes in gut microbial community structure in two-dimensional space. Both temporal gradients and phase-related differences contributed to distinct clustering patterns. The first principal coordinate (PC1) explained 40.15% of the total variance, while the second principal coordinate (PC2) accounted for 29.51%.

**Figure 9:**
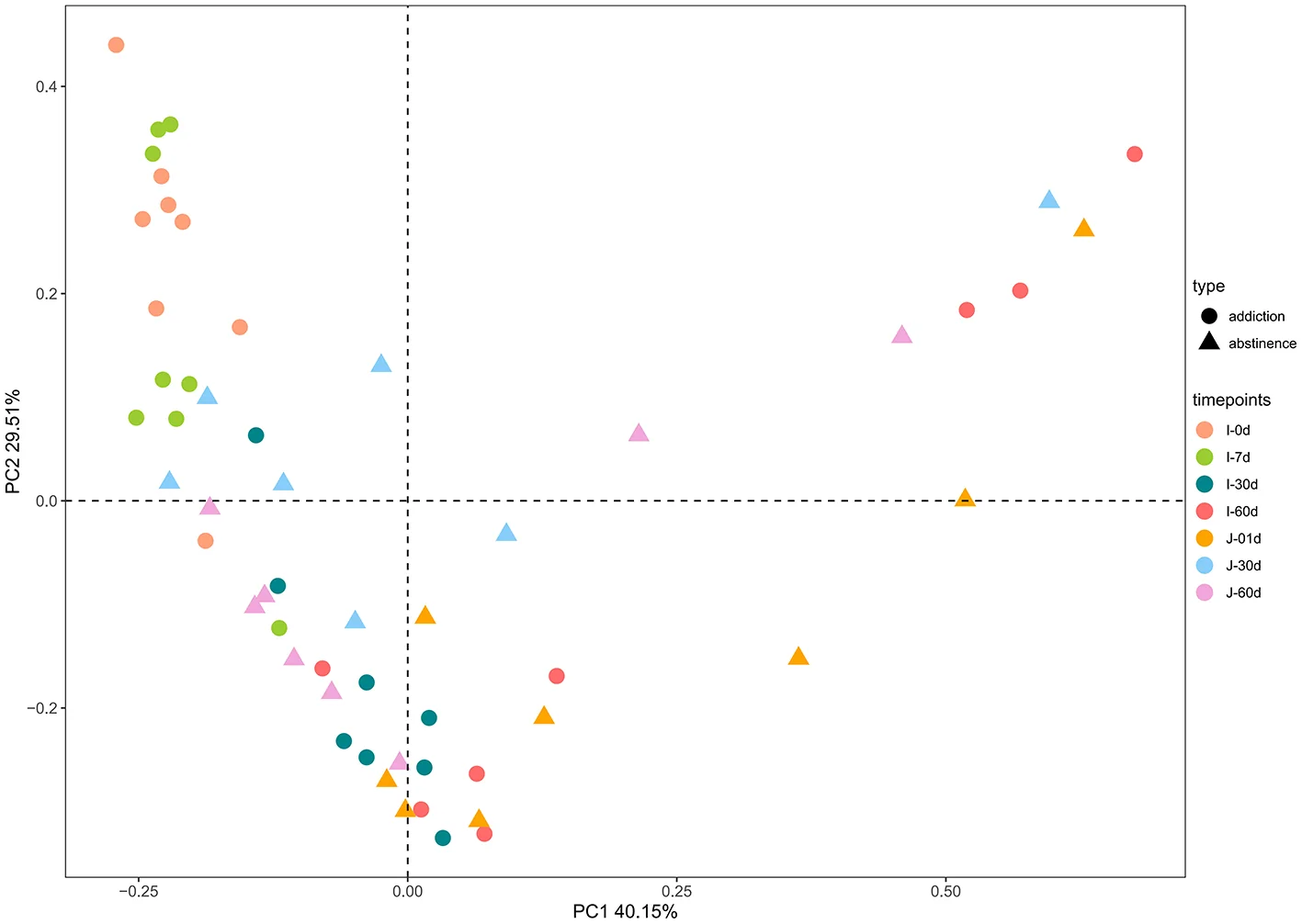
Beta diversity during METH addiction (I) and abstinence (J). Principal coordinates analysis (PCoA) based on Bray–Curtis distances visualizes the dynamic shifts in gut microbial community structure across different time points in two-dimensional space.

The PCoA plot indicated that gut microbial communities exhibited a relatively clustered distribution prior to addiction day 30, followed by pronounced separation at later time points. During the addiction phase, samples from addiction day 0 (baseline) and day 7 were primarily located in the upper-left region of the ordination space and displayed close proximity, forming a compact cluster. Samples from addiction day 30 were mainly distributed in the lower-left region and also showed a tendency toward clustering. During the abstinence phase, samples from abstinence day 30 were concentrated in the central-left region of the ordination space and exhibited a moderate clustering pattern, whereas samples from abstinence day 60 were predominantly distributed in the lower-left region. Permutational multivariate analysis of variance (PERMANOVA) based on Bray–Curtis distances demonstrated that both addiction and abstinence time points had a significant effect on gut microbial community structure (*P* < 0.05).

To further evaluate differences in microbial community structure among specific time points within each phase, pairwise comparisons were performed using the Kruskal–Wallis test (Figure 10). During the addiction phase (Figure 10A), significant differences were observed among most time points (P < 0.05), whereas no significant differences were detected between addiction day 0 and day 7 or between addiction day 7 and day 30 (*P* > 0.05). During the abstinence phase (Figure 10B), no significant difference was observed between abstinence day 30 and day 60, while samples from abstinence day 1 differed significantly from those at other abstinence time points (*P* < 0.05).

**Figure 10:**
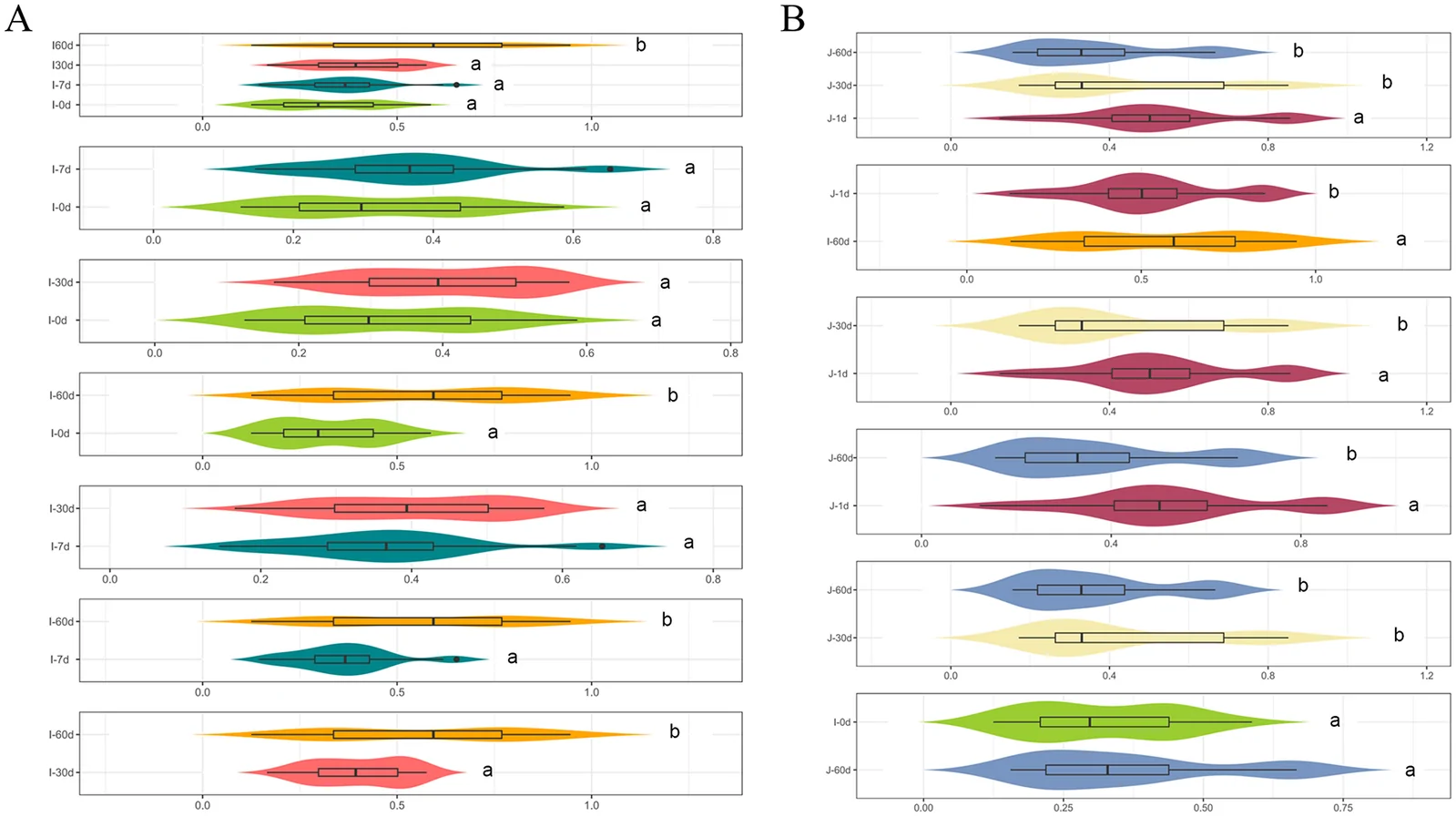
Pairwise comparisons of microbial community structure across different time points during addiction and abstinence phases. Differences in microbial community structure among different time points within the addiction phase **(A)** and abstinence phase **(B)** were evaluated using the Kruskal–Wallis test followed by pairwise comparisons. Different letters indicate statistically significant differences between time points (*P* < 0.05), whereas groups sharing the same letter are not significantly different. The analyses were performed separately within each phase.

#### 3.2.4 Exploratory random forest modeling of temporal gut microbiota dynamics during METH addiction and abstinence

To further explore whether temporal changes in gut microbiota were associated with the duration of METH exposure or abstinence, random forest regression models were constructed separately for the addiction and abstinence phases using genus-level relative abundance data as input variables and addiction/abstinence duration as response variables. For each phase, the dataset was randomly divided into a training set (70%) and a testing set (30%). Model performance was evaluated using the coefficient of determination (*R*^2^), MAE, and RMSE.

During the addiction phase, the random forest model achieved an *R*^2^ of 0.8042 in the training set and 0.7818 in the testing set (Supplementary Table S4), corresponding to approximately 80% and 78% of the variance explained, respectively. The corresponding MAE values were 8.09 days (training) and 9.26 days (testing), and the RMSE values were 9.30 days and 10.40 days, respectively. Given that the addiction phase spanned 60 days, these errors correspond to a relative error of approximately 13%−15%, indicating that genus-level microbial composition captured a substantial proportion of the temporal variation during METH exposure.

For the abstinence phase, the same modeling strategy was applied. The model achieved an *R*^2^ of 0.4808 in the training set and 0.4592 in the testing set, with MAE values of 11.34 days (training) and 13.34 days (testing) and RMSE values of 12.94 and 15.74 days, respectively (Supplementary Table S5). The lower variance explained and larger prediction errors (relative error approximately 19%−22%) suggest increased heterogeneity in gut microbial dynamics during abstinence compared with the addiction phase. This is consistent with the greater dispersion of abstinence samples in the PCoA analysis (Figure 9) and supports the notion that microbial recovery during abstinence is less deterministic.

Because both the temporal prediction and phase classification models were developed and internally evaluated within the same longitudinal cohort, the identified microbial signatures should be considered exploratory rather than validated biomarkers. Independent animal-level validation strategies and external cohorts will be required to determine their robustness and generalizability.

## 4 Discussion

### 4.1 Microbiome-based identification of phase-specific gut microbiota signatures during METH addiction and abstinence

Accumulating evidence highlights the importance of bidirectional interactions between the gut microbiota and the central nervous system in the development and maintenance of addictive behaviors (Salavrakos et al., 2021). As a widely abused central nervous system stimulant, METH has been increasingly linked to dynamic remodeling of the gut microbial community (Miao et al., 2023; Chen et al., 2024). Our findings indicate that distinct alterations in gut microbiota composition occurred during the METH addiction and abstinence phases, suggesting that specific microbial taxa may be associated with different stages of METH exposure.

At the phylum level, *Firmicutes* and *Bacteroidetes* remained the dominant taxa in the Control and Saline groups, accounting for more than 90% of the total microbial abundance, consistent with the core gut microbiota composition of healthy rodents (Chen et al., 2020). In contrast, the relative abundance of *Verrucomicrobiota* exhibited marked fluctuations in METH-treated animals. Previous studies have reported METH-induced disturbances in bile acid metabolism may affect gut microbial composition, and that increased abundance of *Verrucomicrobiaceae* following METH exposure can be attenuated by the bile acid analog obeticholic acid (OCA) (Zhang et al., 2022). *Verrucomicrobiota* have been associated with the regulation of intestinal barrier integrity and inflammatory responses in previous studies (Derrien et al., 2017). The expansion of *Verrucomicrobiota* observed in the present study is therefore consistent with these previously proposed links and raises the possibility that bile acid-related or inflammation-associated pathways may contribute to the microbial remodeling seen during prolonged METH exposure.

At the genus level, the reduced abundance of *Enterococcus* during the METH addiction phase is consistent with its known sensitivity to oxidative stress under intestinal redox conditions (Jose et al., 2018). The increased abundance of *Bifidobacterium* and *Turicibacter* aligns with previously reported functions of these taxa, including short-chain fatty acids (SCFAs) production (Yan et al., 2021) and lipid metabolism (Lynch et al., 2023). Notably, *Desulfovibrio* emerged as a genus distinguishing the addiction and abstinence phases. *Desulfovibrio* participates in sulfate reduction and hydrogen sulfide production, which can influence intestinal barrier function and inflammatory processes in a concentration- and context-dependent manner (Dilek et al., 2020; Ye et al., 2025). The observed reduction in *Desulfovibrio* abundance during METH addiction is therefore consistent with the possibility that METH exposure may interfere with sulfur-related microbial functions. Collectively, these genus-level shifts suggest that METH exposure affects multiple functional guilds within the gut microbiota, potentially through pathways involving oxidative stress, SCFA metabolism, and sulfur cycling—each of which has been independently implicated in gut homeostasis and host–microbe interactions.

These microbial functions may also have potential relevance to the gut–brain axis. SCFAs, particularly acetate, propionate, and butyrate, can influence intestinal barrier integrity, immune signaling, and neuroinflammatory processes, thereby providing potential routes through which gut microbial alterations may affect brain function (Cao et al., 2025; Rosell-Cardona et al., 2025). Similarly, microbial bile acid metabolism can generate signaling molecules that interact with host bile acid receptors and may influence intestinal and systemic metabolic signaling (Chen and Gong, 2025). Sulfur metabolism and hydrogen sulfide production have also been implicated in intestinal barrier function and inflammatory regulation, although their effects may depend on concentration and physiological context (Dilek et al., 2020). Thus, the changes in *Verrucomicrobiota, Bifidobacterium, Turicibacter*, and *Desulfovibrio* observed in the present study may potentially influence gut–brain communication through alterations in microbial metabolites, intestinal barrier function, and immune signaling. However, as we did not measure these intermediate variables directly, our interpretation remains preliminary and warrants caution.

Furthermore, comparative analyses revealed that several genera, including *Ruminococcus, Bacteroides, Akkermansia*, and *Bifidobacterium*, displayed broadly similar abundance trends during METH addiction and abstinence across mouse, rat, and human studies (Deng et al., 2021; Forouzan et al., 2021), although species-specific differences were also evident. Such discrepancies likely reflect interspecies variation in gut microbiota composition and function, differences in experimental design, and host-dependent microbial responses to METH exposure.

α-diversity analysis revealed no significant difference in the Shannon index between the Control and Saline groups, indicating that saline administration alone did not substantially perturb gut microbial diversity. This finding suggests that procedural stress associated with injection had minimal impact on the gut microbiota, consistent with previous reports demonstrating limited effects of handling-related stress in rodent models (Li et al., 2021). In contrast, longitudinal analysis revealed dynamic fluctuations in Shannon diversity during METH addiction and abstinence. Although no significant overall difference was detected across the seven time points, these fluctuations may reflect transient perturbations and subsequent remodeling of the gut microbial community in response to METH exposure and abstinence. This observation is consistent with previous evidence that METH may affect gut microbial homeostasis through multiple mechanisms, including direct effects on SCFA-producing bacteria (Wu et al., 2023) and stress-related hormonal pathways (Wang et al., 2021). The observed increase in diversity during early abstinence may further suggest that these microbial changes are dynamic and at least partially reversible.

Consistent with these findings, β-diversity analysis revealed a clear separation trend in gut microbial community structure between the addiction and abstinence phases. The increased heterogeneity observed during the addiction phase may reflect METH-associated perturbation of the intestinal microbial environment, whereas the relative convergence of microbial communities during abstinence may indicate gradual restoration of host physiological homeostasis. Micke and Novotny similarly observed that opioid withdrawal-induced microbiome alterations were time-dependent and largely resolved during prolonged abstinence, supporting the notion that dynamic microbial remodeling may be a common feature of substance use disorders (Micke and Novotny, 2026).

### 4.2 Microbiome-based analysis of temporal gut microbiota dynamics during METH addiction and abstinence

Temporal analysis of gut microbiota alterations at both the phylum and genus levels revealed that *Verrucomicrobiota* exhibited increased relative abundance during the late phase of METH addiction and throughout the abstinence period. Members of *Verrucomicrobiota* are known to participate in mucin degradation and mucus layer turnover (Zhao et al., 2024; Wang et al., 2025). Their enrichment during late addiction and abstinence may therefore reflect a microbial response to altered mucus dynamics. Butyrate, a metabolite produced by *Firmicutes* and *Verrucomicrobiota*, has been reported to exert context-dependent effects on intestinal epithelium (Kong et al., 2021). The temporal pattern of *Verrucomicrobiota* observed here—rising during late addiction and remaining elevated through abstinence—suggests that this taxon may play a persistent role in the host–microbe interface during and after METH exposure.

At the genus level, four taxa (*Butyricimonas, Candidatus Arthromitus, Enterococcus*, and *Turicibacter*) emerged as key features distinguishing different time points across the addiction and abstinence phases. The dynamic trajectories of these genera highlight phase- and time-associated microbial responses to METH exposure. *Butyricimonas* has been implicated in butyrate production and intestinal barrier function in previous studies (van der Lugt et al., 2019). The gradual increase in *Butyricimonas* abundance over time in the present study aligns with this proposed role and suggests that its trajectory may be relevant to the later phase of addiction and the abstinence period.

*Candidatus Arthromitus* has been associated with intestinal immune regulation, including effects on Treg and Th17 cell populations, in previous work (van de Wouw et al., 2020). The reduced abundance of this genus observed in METH-treated mice is consistent with the hypothesis that METH exposure may affect intestinal immune homeostasis and raises the possibility that immunoregulatory pathways could be involved in the microbial changes seen during addiction.

Alterations in *Enterococcus* abundance may reflect sensitivity to oxidative stress (Jose et al., 2018), as well as reduced competitive fitness during the abstinence phase. In contrast, *Turicibacter* showed reduced abundance during early METH exposure, followed by a gradual increase during abstinence. *Turicibacter* has been linked to lipid metabolism and host energy balance (Lynch et al., 2023), potentially limiting rapid recolonization. In contrast, *Turicibacter* showed reduced abundance during early METH exposure, which may be attributable to direct drug toxicity, acute inflammation, and disruption of the intestinal barrier (Deng et al., 2021). The gradual increase in *Turicibacter* abundance during abstinence may reflect alleviation of toxic stress, immune recovery, and microbiota remodeling mediated by the microbiota–gut–brain axis, including restoration of short-chain fatty acid metabolism and bidirectional neurotransmitter signaling (Fung et al., 2017). Together, these findings underscore the dynamic nature of host–microbiota interactions during the progression and resolution of METH addiction.

α-diversity analyses across multiple time points during METH addiction and abstinence demonstrated that the impact of METH on microbial richness and evenness is time dependent. Notably, a transient increase in the Shannon index was observed at 1 day after abstinence, which may reflect acute abstinence–associated stress responses or rapid compensatory adjustments in neurotransmitter systems, such as dopamine signaling (Rusyniak, 2011; Chartoff and Carlezon, 2014). Previous studies have reported elevated levels of brain-derived neurotrophic factor (BDNF) during early abstinence (1–7 days), followed by a subsequent decline (Razavi et al., 2022). Given the reported associations between BDNF dynamics and gut microbiota alterations, fluctuations in this neuroplasticity-related factor may be linked to the observed temporal changes in microbial diversity.

Previous studies have reported phase-dependent neuroinflammatory responses to METH (Mai et al., 2024), and the gut microbiota is known to interact with neuroinflammatory processes through various pathways (Cavaliere and Traina, 2023; Warren et al., 2024). The phase-specific microbial changes observed in our study are consistent with the possibility that neuroinflammatory dynamics may influence gut microbial communities during different stages of the addiction cycle. This convergence of microbial and neuroinflammatory patterns across phases suggests that the gut–brain axis may be particularly relevant to understanding the temporal progression of METH-related pathology.

Consistent with these observations, principal coordinates analysis (PCoA) revealed distinct temporal patterns in sample distribution across addiction and abstinence phases. Clustering of samples at addiction day 0 and addiction day 7 suggests microbial adaptation or transient stabilization under short-term METH exposure, whereas the dispersion of samples during abstinence may reflect progressive host recovery and microbiota remodeling.

Exploratory random forest analyses further supported these phase-dependent microbial dynamics. The temporal prediction model showed higher performance during the addiction phase than during abstinence, whereas the reduced modeling performance during abstinence may reflect greater variability in microbiota remodeling during recovery. These findings, together with the greater dispersion observed in PCoA, suggest that gut microbial changes during abstinence are more variable than those during active METH exposure.

Because these machine-learning models were developed and internally evaluated within the same longitudinal cohort, the identified microbial signatures should be considered exploratory rather than validated biomarkers. Independent animal-level validation and external cohorts will be required to determine their robustness and generalizability.

The present study provides a longitudinal taxonomic framework for gut microbiota remodeling during METH addiction and abstinence. However, the use of 16S rRNA sequencing and relatively sparse sampling during abstinence limited taxonomic and temporal resolution, while microbial metabolites, inflammatory responses, and gut–brain-related functional endpoints were not directly assessed. Future studies incorporating higher-resolution microbiome profiling, denser longitudinal sampling, and functional validation will help clarify the biological relevance of these microbial changes.

## 5 Conclusion

This study used longitudinal microbiome analyses to characterize phase-specific differences and temporal dynamics of gut microbiota during METH addiction and abstinence. The addiction and abstinence phases showed significant differences in microbial composition, α- and β-diversity, and overall community structure. Community dispersion was higher during the addiction phase, whereas microbial communities became progressively more stable during abstinence. Time-series analysis identified shifts in *Verrucomicrobia* and *Actinobacteria* during prolonged METH exposure and early abstinence. At the genus level, *Butyricimonas, Candidatus Arthromitus, Enterococcus*, and *Turicibacter* displayed consistent phase-associated and time-dependent patterns. Exploratory classification and temporal prediction models based on these features showed higher performance during the addiction phase but lower performance during abstinence, consistent with greater microbial heterogeneity during abstinence. Collectively, these findings provide longitudinal evidence of distinct and dynamic gut microbiota remodeling across METH addiction and abstinence and provide a basis for further investigation of microbiota-associated signatures and their potential relevance to METH addiction and recovery. However, the identified microbial signatures remain exploratory and require validation in larger cohorts and at the animal level, together with functional studies to determine their biological relevance.

## Data Availability

The original contributions presented in the study are included in the article/Supplementary material, further inquiries can be directed to the corresponding author/s.
