# Supplementary material for: Longitudinal dynamics of the gut microbiome during methamphetamine addiction and abstinence
Source: Front Microbiol. 2026 Sep 23;17:1911776. doi: 10.3389/fmicb.2026.1911776 (PMC13644584; doi:10.3389/fmicb.2026.1911776)
Supplement: Supplementary file 1 [file Supplementary_file_1.docx]

**Supplementary Materials for**

**Longitudinal Dynamics of the Gut Microbiome during Methamphetamine** **Addiction and Abstinence**

Lingxiao Wang^1^ · Jiaqi Wang^1^ · Yi Zhang^1^ · Baojing Wang^1^· Xiaomeng Sun^2^ · Yanhua Li^3^ · Jiangwei Yan^1,4,*^ · Keming Yun^1,4,*^ · Hongliang Su^1,4,*^

^1^ School of Forensic Medicine, Shanxi Medical University, Taiyuan 030001, PR China

^2^ Technical Appraisal Research Center of the People’s Procuratorate of Shanxi Province, Taiyuan 030001, PR China

^3^ Department of Foreign Languages, Shanxi Medical University, Taiyuan 030001, PR China

^4^ Key Laboratory of Forensic Toxicology, Ministry of Public Security, Beijing 100192, PR China

^*^ Corresponding authors

Jiangwei Yan, Keming Yun and Hongliang Su

Jiangwei Yan, E-mail address: yanjw@sxmu.edu.cn

Keming Yun, E-mail address: yunkeming5142@163.com

Hongliang Su, E-mail address: hongliangsu@sxmu.edu.cn

Lingxiao Wang and Jiaqi Wang have equal contribution.

Key words methamphetamine, gut microbiota, addiction, abstinence, machine learning

**This file includes:**

**Figs. S1 to S3**

**Tables S1 to S5**

**Figure S1.** **Rarefaction curves.** Curves showing the sequencing depth saturation after quality control and normalization. The x-axis represents the sequencing depth (number of reads subsampled per sample), and the y-axis represents the observed species richness (OTUs). Each colored curve represents an individual sample (total n = 159). All samples were rarefied to the same sequencing depth prior to analysis. The plateauing of all curves at the final sequencing depth indicates that the microbial diversity in each sample was adequately captured. Blue curves, Addiction group; red curves, Abstinence group.

**Supplementary Figure 2. Identification of differential taxa by LEfSe analysis.Linear discriminant analysis Effect Size (LEfSe) was performed to identify bacterial taxa that were differentially abundant between the METH addiction phase and the withdrawal phase. At the genus level, *Dehalobacterium* was identified as a key discriminative taxon. The median relative abundance of *Dehalobacterium* was consistently lower in the METH group, whereas the non_METH group exhibited a higher median with a broader distribution, indicating greater variability. This suggests that *Dehalobacterium* may be more prevalent or diverse in the non_METH condition.**

**Supplementary Figure 3. The relative abundance of the gut microbiota in the METH group.** Stacked bar charts show the composition at the phylum (A) and genus (B) levels in mice fecal samples at different time points during the METH addiction (I) and abstinence (J) phases.

**Supplementary Table 1.** The raw reads detail of the sequenced samples.

| **Sample Name** | **Reads Counds** | **Sample Name** | **Reads Counds** | **Sample Name** | **Reads Counds** |
| --- | --- | --- | --- | --- | --- |
| I30M9 | 4285 | I7C12 | 18133 | I7C5 | 32668 |
| J1S12 | 4483 | J60S7 | 18472 | J60M9 | 32720 |
| I60C6 | 4931 | I30S8 | 18746 | I60M5 | 33440 |
| J30C7 | 5640 | J30S6 | 18805 | J60S1 | 33528 |
| J30C12 | 5965 | I30M7 | 18888 | J1M9 | 33560 |
| J30M12 | 6636 | J30S8 | 18997 | J1C3 | 34403 |
| I30M8 | 6728 | I7C6 | 19217 | J30C9 | 34893 |
| I60S3 | 7222 | I30S12 | 19774 | I60M3 | 34981 |
| I30C7 | 7343 | I30C6 | 19991 | I60M7 | 35779 |
| J30S7 | 7476 | I0S3 | 20039 | J30C3 | 36451 |
| J1S7 | 7570 | J60S12 | 20163 | I0S6 | 37041 |
| I30S7 | 8816 | I60C3 | 20325 | J30C5 | 37289 |
| I60M9 | 9069 | I60M8 | 20904 | J60S5 | 38371 |
| J30M6 | 9458 | I0M3 | 21436 | I30S3 | 38680 |
| I30C12 | 9686 | J60M7 | 21577 | J60M5 | 38942 |
| I0M8 | 10150 | I0C12 | 22121 | J1S3 | 39368 |
| I7M8 | 10622 | J30S3 | 22206 | I60M1 | 40194 |
| J1S6 | 10842 | J60C1 | 22376 | J1C9 | 41559 |
| I7S3 | 10895 | J60M6 | 22442 | I0C6 | 41802 |
| I7S8 | 11086 | I7M7 | 22534 | J1S8 | 42330 |
| I60S5 | 11149 | I0M12 | 22538 | I30C8 | 42405 |
| J1C12 | 11193 | I30M1 | 22578 | J60S8 | 42622 |
| J30C6 | 11259 | J1M6 | 22670 | J1C8 | 42919 |
| I60S12 | 11590 | I0C1 | 22802 | J30M5 | 42954 |
| I0C7 | 11843 | I7M1 | 23010 | J30M3 | 43387 |
| I7M12 | 12295 | I7M5 | 23135 | I30C9 | 43918 |
| J30S1 | 12446 | J1S1 | 23238 | I0M1 | 44448 |
| J1C1 | 12646 | I7S7 | 23681 | J60S3 | 45567 |
| I60C12 | 12663 | I7C1 | 23987 | J60C5 | 46253 |
| I7C8 | 12935 | I0C8 | 24833 | J30C8 | 47196 |
| J60C6 | 13302 | J60M1 | 24846 | I0C9 | 47684 |
| I30M5 | 13496 | I30M12 | 25084 | I0C5 | 47998 |
| I60C7 | 14041 | J1C7 | 25109 | J60M3 | 48355 |
| J60C12 | 14132 | I30M6 | 25736 | I0M7 | 49866 |
| J1C6 | 14407 | J1M7 | 25762 | J30M9 | 52799 |
| J60C7 | 14413 | I7M6 | 26277 | I30C5 | 52943 |
| I60C9 | 14821 | J30M1 | 26308 | J30M8 | 53565 |
| I0S9 | 14921 | J60M12 | 27237 | J1S5 | 54986 |
| I60M12 | 15015 | I7C3 | 27245 | J1M3 | 55699 |
| I30M3 | 15053 | I30C1 | 27566 | I30C3 | 56398 |
| J30C1 | 15193 | I7M3 | 27628 | J1M8 | 56988 |
| J30S9 | 15198 | I7C9 | 27710 | J60C3 | 57230 |
| I0S8 | 15234 | I7S6 | 27926 | I60M6 | 57567 |
| J1M12 | 15250 | J60S9 | 28620 | J1S9 | 60077 |
| J1M1 | 15583 | I30S6 | 28718 | I0C3 | 61126 |
| I0S7 | 16137 | I0S12 | 28722 | J30S5 | 66372 |
| I60C8 | 16332 | I0M5 | 28959 | J60M8 | 72148 |
| I7C7 | 16586 | J1M5 | 28973 | J60C9 | 75069 |
| I7S9 | 16713 | J60S6 | 29037 | J1C5 | 75517 |
| I30S1 | 16751 | I7S12 | 29467 |  |  |
| I60S1 | 16764 | J60C8 | 29509 |  |  |
| I7M9 | 16847 | I30S5 | 31176 |  |  |
| I60S9 | 17088 | I0S5 | 31440 |  |  |
| I60C5 | 17107 | I0M6 | 32180 |  |  |
| I30S9 | 17597 | I0M9 | 32444 |  |  |

**Supplementary Table 2.** The abundance of bacteria phylum in mice with different timepoint and different group in this study.

| **Phylum** | **Addiction (abundance %)** | | | | | | | | | | | |
| --- | --- | --- | --- | --- | --- | --- | --- | --- | --- | --- | --- | --- |
|  | **0d** | | | **7d** | | | **30d** | | | **60d** | | |
|  | **C** | **S** | **M** | **C** | **S** | **M** | **C** | **S** | **M** | **C** | **S** | **M** |
| p__Actinobacteria | 0.9772 | 0.8735 | 0.8139 | 0.6126 | 0.7079 | 0.8839 | 0.7497 | 0.6768 | 16.6657 | 0.5834 | 2.1144 | 7.4008 |
| p__Bacteroidetes | 27.5583 | 26.1577 | 21.2544 | 39.2649 | 26.7872 | 27.6342 | 53.7077 | 56.1260 | 43.7340 | 60.6834 | 49.4096 | 27.6809 |
| p__Chloroflexi | 0.0000 | 0.0000 | 0.0000 | 0.0029 | 0.0000 | 0.0000 | 0.0000 | 0.0058 | 0.0000 | 0.0000 | 0.0047 | 0.0000 |
| p__Cyanobacteria | 0.3092 | 0.1600 | 0.3938 | 0.9802 | 0.0389 | 0.4113 | 1.1989 | 0.2013 | 0.2421 | 0.2967 | 0.4901 | 0.2859 |
| p__Deferribacteres | 0.1984 | 0.0467 | 0.3005 | 0.1721 | 0.1322 | 0.1021 | 0.0817 | 0.1138 | 0.0496 | 0.0933 | 0.0607 | 0.0029 |
| p__Firmicutes | 69.7841 | 71.5719 | 76.3477 | 57.7392 | 71.1319 | 66.3740 | 43.3343 | 41.9691 | 37.5963 | 36.5928 | 46.5298 | 32.5554 |
| p__Fusobacteria | 0.0029 | 0.0067 | 0.0000 | 0.0088 | 0.0078 | 0.01 | 0.0000 | 0.0029 | 0.0000 | 0.0000 | 0.0000 | 0.0000 |
| p__Gemmatimonadetes | 0.0000 | 0.0000 | 0.0000 | 0.0000 | 0.0000 | 0.0058 | 0.0000 | 0.0000 | 0.0000 | 0.0000 | 0.0000 | 0.0000 |
| p__Proteobacteria | 1.1348 | 1.1302 | 0.8576 | 1.2019 | 1.1746 | 4.5566 | 0.9218 | 0.8810 | 0.9160 | 1.7203 | 1.3816 | 0.7847 |
| p__Spirochaetes | 0.0000 | 0.0000 | 0.0029 | 0.0000 | 0.0000 | 0.0000 | 0.0000 | 0.0000 | 0.0000 | 0.0000 | 0.0000 | 0.0000 |
| p__Synergistetes | 0.0029 | 0.0000 | 0.0000 | 0.0000 | 0.0000 | 0.0029 | 0.0000 | 0.0000 | 0.0000 | 0.0000 | 0.0000 | 0.0000 |
| p__Tenericutes | 0.0000 | 0.0433 | 0.0029 | 0.0029 | 0.0000 | 0.0000 | 0.0000 | 0.0088 | 0.0029 | 0.0000 | 0.0000 | 0.0000 |
| p__TM7 | 0.0233 | 0.0100 | 0.0146 | 0.0146 | 0.0078 | 0.0058 | 0.0058 | 0.0146 | 0.0058 | 0.0067 | 0.0000 | 0.0000 |
| p__Verrucomicrobia | 0.0088 | 0.0000 | 0.0117 | 0.0000 | 0.0117 | 0.0117 | 0.0000 | 0.0000 | 0.7876 | 0.0233 | 0.0093 | 31.2894 |

**Supplementary Table 2** **continued.** The abundance of bacteria phylum in mice with different timepoint and different group in this study.

| **Phylum** | **Abstinence (abundance %)** | | | | | | | | |
| --- | --- | --- | --- | --- | --- | --- | --- | --- | --- |
|  | **1d** | | | **30d** | | | **60d** | | |
|  | **C** | **S** | **M** | **C** | **S** | **M** | **C** | **S** | **M** |
| p__Actinobacteria | 3.0368 | 2.5642 | 10.8110 | 3.3460 | 0.9368 | 0.6068 | 3.7515 | 1.1464 | 0.2567 |
| p__Bacteroidetes | 51.0473 | 57.2637 | 29.5333 | 45.6418 | 35.0492 | 39.3066 | 46.5373 | 41.0589 | 54.6412 |
| p__Chloroflexi | 0.0000 | 0.0000 | 0.0029 | 0.0000 | 0.0000 | 0.0000 | 0.0000 | 0.0000 | 0.0000 |
| p__Cyanobacteria | 0.5630 | 0.9452 | 0.4347 | 0.4463 | 0.6468 | 1.0035 | 0.2917 | 1.0152 | 0.4988 |
| p__Deferribacteres | 0.0525 | 0.0233 | 0.0233 | 0.0379 | 0.0500 | 0.1000 | 0.0817 | 0.0875 | 0.1313 |
| p__Firmicutes | 43.7894 | 37.9842 | 37.8763 | 45.3005 | 60.2400 | 39.3266 | 38.5648 | 50.6943 | 31.2223 |
| p__Fusobacteria | 0.0029 | 0.0088 | 0.0000 | 0.0000 | 0.0000 | 0.0000 | 0.0029 | 0.0000 | 0.0000 |
| p__Gemmatimonadetes | 0.0000 | 0.0000 | 0.0000 | 0.0000 | 0.0000 | 0.0000 | 0.0000 | 0.0000 | 0.0000 |
| p__Proteobacteria | 1.4994 | 1.1406 | 1.1989 | 0.8985 | 0.7235 | 0.9602 | 1.5578 | 0.6009 | 0.9277 |
| p__Spirochaetes | 0.0000 | 0.0000 | 0.0000 | 0.0000 | 0.0000 | 0.0033 | 0.0000 | 0.0000 | 0.0000 |
| p__Synergistetes | 0.0000 | 0.0000 | 0.0000 | 0.0000 | 0.0000 | 0.0000 | 0.0000 | 0.0000 | 0.0000 |
| p__Tenericutes | 0.0000 | 0.0058 | 0.0000 | 0.0000 | 0.0000 | 0.0000 | 0.0088 | 0.0000 | 0.0000 |
| p__TM7 | 0.0000 | 0.0204 | 0.0058 | 0.0000 | 0.0033 | 0.0067 | 0.0029 | 0.0000 | 0.0029 |
| p__Verrucomicrobia | 0.0088 | 0.0438 | 20.1138 | 4.3291 | 2.3504 | 18.6864 | 9.2007 | 5.3967 | 12.3191 |

**Supplementary Table 3.** The abundance of bacteria genus in mice with different timepoint and different group in this study.

| **Genus** | **Addiction (abundance %)** | | | | | | | | | | | | |
| --- | --- | --- | --- | --- | --- | --- | --- | --- | --- | --- | --- | --- | --- |
|  | **0d** | | | **7d** | | | **30d** | | | **60d** | | | |
|  | **C** | **S** | **M** | **C** | **S** | **M** | **C** | **S** | **M** | **C** | **S** | **M** |  |
| g__[Prevotella] | 0.7900 | 0.2400 | 1.3100 | 0.5718 | 0.3228 | 0.2713 | 1.3361 | 0.6914 | 0.6039 | 0.2967 | 0.4434 | 0.2655 |  |
| g__[Ruminococcus] | 0.8100 | 1.4700 | 1.4700 | 0.5018 | 0.9607 | 0.9918 | 0.8722 | 0.4347 | 0.1984 | 0.4134 | 0.4341 | 0.1546 |  |
| g__02d06 | 0.0000 | 0.0000 | 0.0000 | 0.0000 | 0.0000 | 0.0000 | 0.0000 | 0.0000 | 0.0000 | 0.0000 | 0.0000 | 0.0058 |  |
| g__Acinetobacter | 0.0000 | 0.0000 | 0.0000 | 0.0000 | 0.0000 | 0.0000 | 0.0000 | 0.0000 | 0.0000 | 0.0000 | 0.0000 | 0.0000 |  |
| g__Actinomyces | 0.0000 | 0.0030 | 0.0000 | 0.0000 | 0.0039 | 0.0029 | 0.0029 | 0.0000 | 0.0000 | 0.0000 | 0.0000 | 0.0000 |  |
| g__Actinopolymorpha | 0.0000 | 0.0000 | 0.0000 | 0.0000 | 0.0000 | 0.0000 | 0.0000 | 0.0029 | 0.0000 | 0.0000 | 0.0000 | 0.0000 |  |
| g__Adlercreutzia | 0.9500 | 0.8500 | 0.7900 | 0.5980 | 0.6768 | 0.8285 | 0.6009 | 0.6214 | 0.6214 | 0.2300 | 0.3781 | 0.1313 |  |
| g__Aggregatibacter | 0.0000 | 0.0000 | 0.0000 | 0.0000 | 0.0000 | 0.0029 | 0.0000 | 0.0000 | 0.0000 | 0.0000 | 0.0000 | 0.0000 |  |
| g__Akkermansia | 0.0100 | 0.0000 | 0.0120 | 0.0000 | 0.0117 | 0.0117 | 0.0000 | 0.0000 | 0.7876 | 0.0233 | 0.0093 | 31.2894 |  |
| g__Alistipes | 0.0000 | 0.0000 | 0.0000 | 0.0029 | 0.0000 | 0.0029 | 0.0029 | 0.0000 | 0.0058 | 0.0100 | 0.0047 | 0.0029 |  |
| g__Allobaculum | 0.0000 | 0.0030 | 0.0000 | 0.0000 | 0.0000 | 0.0000 | 0.0088 | 0.0175 | 1.0035 | 0.3234 | 0.0607 | 0.0671 |  |
| g__Anaerofustis | 0.0000 | 0.0000 | 0.0000 | 0.0029 | 0.0000 | 0.0000 | 0.0000 | 0.0000 | 0.0000 | 0.0000 | 0.0000 | 0.0000 |  |
| g__Anaeroplasma | 0.0000 | 0.0400 | 0.0030 | 0.0000 | 0.0000 | 0.0000 | 0.0000 | 0.0088 | 0.0000 | 0.0000 | 0.0000 | 0.0000 |  |
| g__Anaerostipes | 0.0000 | 0.0030 | 0.0000 | 0.0000 | 0.0000 | 0.0000 | 0.0000 | 0.0029 | 0.0000 | 0.0033 | 0.0140 | 0.0000 |  |
| g__Anaerotruncus | 0.0030 | 0.0000 | 0.0000 | 0.0000 | 0.0000 | 0.0029 | 0.0000 | 0.0000 | 0.0000 | 0.0000 | 0.0000 | 0.0000 |  |
| g__Anaerovorax | 0.0000 | 0.0000 | 0.0000 | 0.0000 | 0.0000 | 0.0029 | 0.0000 | 0.0000 | 0.0000 | 0.0000 | 0.0000 | 0.0000 |  |
| g__Arthrobacter | 0.0000 | 0.0000 | 0.0000 | 0.0000 | 0.0000 | 0.0029 | 0.0000 | 0.0000 | 0.0000 | 0.0000 | 0.0000 | 0.0000 |  |
| g__Atopobium | 0.0000 | 0.0000 | 0.0000 | 0.0000 | 0.0000 | 0.0000 | 0.0000 | 0.0029 | 0.0000 | 0.0000 | 0.0000 | 0.0000 |  |
| g__Bacillus | 0.0000 | 0.0000 | 0.0000 | 0.0000 | 0.0000 | 0.0000 | 0.0029 | 0.0000 | 0.0000 | 0.0000 | 0.0047 | 0.0000 |  |

**Supplementary Table 3 continued.** The abundance of bacteria genus in mice with different timepoint and different group in this study.

| **Genus** | **Addiction (abundance %)** | | | | | | | | | | | | |
| --- | --- | --- | --- | --- | --- | --- | --- | --- | --- | --- | --- | --- | --- |
|  | **0d** | | | **7d** | | | **30d** | | | **60d** | | | |
|  | **C** | **S** | **M** | **C** | **S** | **M** | **C** | **S** | **M** | **C** | **S** | **M** |  |
| g__Bacteroides | 1.2800 | 0.8700 | 1.3600 | 0.9102 | 1.6958 | 0.3559 | 1.4615 | 1.5082 | 1.7328 | 1.0735 | 1.7923 | 0.6651 |  |
| g__Bifidobacterium | 0.0100 | 0.0000 | 0.0000 | 0.0029 | 0.0156 | 0.0029 | 0.1109 | 0.0058 | 16.0414 | 0.3434 | 1.7316 | 7.2608 |  |
| g__Bilophila | 0.1700 | 0.1800 | 0.2700 | 0.1867 | 0.2061 | 0.1284 | 0.1225 | 0.1604 | 0.1138 | 0.1700 | 0.1400 | 0.0233 |  |
| g__Blautia | 0.0030 | 0.0000 | 0.0000 | 0.0000 | 0.0039 | 0.0000 | 0.0000 | 0.0000 | 0.0000 | 0.0000 | 0.0000 | 0.0000 |  |
| g__Butyricimonas | 0.0500 | 0.0400 | 0.0500 | 0.0496 | 0.0428 | 0.1313 | 0.1254 | 0.0963 | 0.0788 | 0.3934 | 0.2520 | 0.1488 |  |
| g__Candidatus Arthromitus | 0.9200 | 2.4600 | 1.7300 | 0.2596 | 0.3501 | 0.2392 | 0.1604 | 0.1400 | 0.0467 | 0.0133 | 0.0093 | 0.0000 |  |
| g__Capnocytophaga | 0.0000 | 0.0000 | 0.0000 | 0.0029 | 0.0000 | 0.0000 | 0.0000 | 0.0000 | 0.0000 | 0.0000 | 0.0000 | 0.0000 |  |
| g__Catonella | 0.0000 | 0.0000 | 0.0000 | 0.0000 | 0.0000 | 0.0000 | 0.0000 | 0.0000 | 0.0000 | 0.0000 | 0.0000 | 0.0000 |  |
| g__Citrobacter | 0.0000 | 0.0000 | 0.0000 | 0.0000 | 0.0000 | 0.0029 | 0.0000 | 0.0000 | 0.0000 | 0.0000 | 0.0000 | 0.0000 |  |
| g__Clostridium | 0.0100 | 0.0270 | 0.0120 | 0.0350 | 0.0000 | 0.0029 | 0.0088 | 0.0088 | 0.0058 | 0.0000 | 0.0000 | 0.0117 |  |
| g__Cohnella | 0.0000 | 0.0030 | 0.0000 | 0.0000 | 0.0000 | 0.0000 | 0.0000 | 0.0000 | 0.0000 | 0.0000 | 0.0000 | 0.0000 |  |
| g__Coprobacillus | 0.0500 | 0.0430 | 0.0300 | 0.0321 | 0.0117 | 0.0554 | 0.0583 | 0.1138 | 0.0204 | 0.0633 | 0.0327 | 0.0146 |  |
| g__Coprococcus | 0.2500 | 0.3200 | 0.2000 | 0.0992 | 0.1011 | 0.1371 | 0.3646 | 0.1867 | 0.1721 | 0.1034 | 0.4014 | 0.2334 |  |
| g__Corynebacterium | 0.0000 | 0.0030 | 0.0030 | 0.0000 | 0.0000 | 0.0000 | 0.0000 | 0.0000 | 0.0000 | 0.0000 | 0.0000 | 0.0000 |  |
| g__Dehalobacterium | 0.1100 | 0.0500 | 0.1000 | 0.1371 | 0.1011 | 0.0788 | 0.1284 | 0.1517 | 0.0933 | 0.1934 | 0.1354 | 0.0117 |  |
| g__Dialister | 0.0000 | 0.0000 | 0.0030 | 0.0000 | 0.0000 | 0.0000 | 0.0000 | 0.0000 | 0.0000 | 0.0000 | 0.0000 | 0.0000 |  |
| g__Dorea | 0.1100 | 0.2600 | 0.2400 | 0.0875 | 0.1206 | 0.2130 | 0.0729 | 0.0408 | 0.1225 | 0.1134 | 0.0467 | 0.0758 |  |
| g__Enterococcus | 14.1400 | 6.6500 | 4.9100 | 8.3576 | 8.5375 | 2.3600 | 6.5723 | 0.0088 | 0.0846 | 0.0200 | 0.0280 | 0.0000 |  |
| g__Erwinia | 0.0010 | 0.0000 | 0.0000 | 0.0029 | 0.0000 | 0.0000 | 0.0000 | 0.0000 | 0.0000 | 0.0000 | 0.0000 | 0.0000 |  |

**Supplementary Table 3 continued.** The abundance of bacteria genus in mice with different timepoint and different group in this study.

| **Genus** | **Addiction (abundance %)** | | | | | | | | | | | | |
| --- | --- | --- | --- | --- | --- | --- | --- | --- | --- | --- | --- | --- | --- |
|  | **0d** | | | **7d** | | | **30d** | | | **60d** | | | |
|  | **C** | **S** | **M** | **C** | **S** | **M** | **C** | **S** | **M** | **C** | **S** | **M** |  |
| g__Exiguobacterium | 0.0000 | 0.0000 | 0.0000 | 0.0000 | 0.0000 | 0.0000 | 0.0000 | 0.0000 | 0.0000 | 0.0000 | 0.0000 | 0.0000 |  |
| g__Flavisolibacter | 0.0000 | 0.0000 | 0.0000 | 0.0000 | 0.0000 | 0.0000 | 0.0000 | 0.0000 | 0.0000 | 0.0000 | 0.0000 | 0.0029 |  |
| g__Flavobacterium | 0.0000 | 0.0000 | 0.0000 | 0.0000 | 0.0000 | 0.0000 | 0.0029 | 0.0029 | 0.0000 | 0.0000 | 0.0000 | 0.0000 |  |
| g__Fusobacterium | 0.0000 | 0.0000 | 0.0000 | 0.0058 | 0.0078 | 0.0058 | 0.0000 | 0.0029 | 0.0000 | 0.0000 | 0.0000 | 0.0000 |  |
| g__Gemella | 0.0000 | 0.0000 | 0.0000 | 0.0000 | 0.0000 | 0.0000 | 0.0000 | 0.0000 | 0.0000 | 0.0000 | 0.0000 | 0.0000 |  |
| g__Geosporobacter_Thermotalea | 0.0000 | 0.0000 | 0.0000 | 0.0000 | 0.0000 | 0.0000 | 0.0029 | 0.0000 | 0.0000 | 0.0000 | 0.0000 | 0.0000 |  |
| g__Granulicatella | 0.0000 | 0.0030 | 0.0030 | 0.0029 | 0.0078 | 0.0029 | 0.0029 | 0.0029 | 0.0029 | 0.0000 | 0.0000 | 0.0000 |  |
| g__Haemophilus | 0.0000 | 0.0000 | 0.0030 | 0.0000 | 0.0000 | 0.0000 | 0.0000 | 0.0000 | 0.0000 | 0.0000 | 0.0000 | 0.0029 |  |
| g__Janthinobacterium | 0.0000 | 0.0000 | 0.0000 | 0.0000 | 0.0039 | 0.0000 | 0.0000 | 0.0000 | 0.0000 | 0.0000 | 0.0000 | 0.0000 |  |
| g__Kaistobacter | 0.0000 | 0.0000 | 0.0000 | 0.0000 | 0.0000 | 0.0000 | 0.0000 | 0.0000 | 0.0000 | 0.0000 | 0.0000 | 0.0000 |  |
| g__Klebsiella | 0.0100 | 0.0000 | 0.0000 | 0.0000 | 0.0000 | 0.0029 | 0.0000 | 0.0000 | 0.0000 | 0.0067 | 0.0000 | 0.0000 |  |
| g__Kocuria | 0.0000 | 0.0000 | 0.0000 | 0.0000 | 0.0000 | 0.0000 | 0.0000 | 0.0000 | 0.0000 | 0.0000 | 0.0000 | 0.0000 |  |
| g__Lachnobacterium | 0.0000 | 0.0100 | 0.0000 | 0.0000 | 0.0233 | 0.0000 | 0.0000 | 0.0000 | 0.0000 | 0.0000 | 0.0000 | 0.0000 |  |
| g__Lactobacillus | 1.7600 | 4.1800 | 2.4100 | 2.0595 | 1.0735 | 1.6394 | 3.9819 | 2.0041 | 10.7935 | 2.6138 | 1.4516 | 14.2445 |  |
| g__Lactococcus | 0.0000 | 0.0000 | 0.0000 | 0.0000 | 0.0000 | 0.0029 | 0.0029 | 0.0000 | 0.0000 | 0.0000 | 0.0000 | 0.0000 |  |
| g__Lautropia | 0.0000 | 0.0000 | 0.0000 | 0.0000 | 0.0039 | 0.0029 | 0.0029 | 0.0000 | 0.0000 | 0.0033 | 0.0000 | 0.0000 |  |
| g__Leptotrichia | 0.0030 | 0.0100 | 0.0000 | 0.0029 | 0.0000 | 0.0058 | 0.0000 | 0.0000 | 0.0000 | 0.0000 | 0.0000 | 0.0000 |  |
| g__Luteimonas | 0.0000 | 0.0000 | 0.0000 | 0.0000 | 0.0000 | 0.0000 | 0.0000 | 0.0029 | 0.0000 | 0.0000 | 0.0000 | 0.0000 |  |
| g__Megasphaera | 0.0000 | 0.0000 | 0.0000 | 0.0000 | 0.0000 | 0.0029 | 0.0000 | 0.0029 | 0.0000 | 0.0000 | 0.0000 | 0.0000 |  |

**Supplementary Table 3 continued.** The abundance of bacteria genus in mice with different timepoint and different group in this study.

| **Genus** | **Addiction (abundance %)** | | | | | | | | | | | | |
| --- | --- | --- | --- | --- | --- | --- | --- | --- | --- | --- | --- | --- | --- |
|  | **0d** | | | **7d** | | | **30d** | | | **60d** | | | |
|  | **C** | **S** | **M** | **C** | **S** | **M** | **C** | **S** | **M** | **C** | **S** | **M** |  |
| g__Morganella | 0.0000 | 0.0000 | 0.0000 | 0.0000 | 0.0000 | 0.0000 | 0.0000 | 0.0000 | 0.0000 | 0.0000 | 0.0000 | 0.0000 |  |
| g__Moryella | 0.0000 | 0.0000 | 0.0000 | 0.0000 | 0.0000 | 0.0029 | 0.0000 | 0.0000 | 0.0000 | 0.0000 | 0.0000 | 0.0000 |  |
| g__Mucispirillum | 0.2000 | 0.0500 | 0.3000 | 0.1721 | 0.1322 | 0.1021 | 0.0817 | 0.1138 | 0.0496 | 0.0933 | 0.0607 | 0.0029 |  |
| g__Neisseria | 0.0100 | 0.0100 | 0.0030 | 0.0029 | 0.0156 | 0.0058 | 0.0000 | 0.0088 | 0.0000 | 0.0000 | 0.0000 | 0.0000 |  |
| g__Odoribacter | 0.2400 | 0.2100 | 0.3300 | 0.2159 | 0.2606 | 0.2975 | 0.4988 | 0.3121 | 0.3092 | 0.1600 | 0.2660 | 0.1313 |  |
| g__Oribacterium | 0.0100 | 0.0000 | 0.0000 | 0.0000 | 0.0000 | 0.0000 | 0.0000 | 0.0000 | 0.0000 | 0.0000 | 0.0000 | 0.0000 |  |
| g__Oscillospira | 1.7400 | 1.1400 | 1.8600 | 1.6978 | 1.2174 | 1.9137 | 2.3950 | 1.4907 | 1.0035 | 3.5806 | 1.7876 | 0.8401 |  |
| g__Paenibacillus | 0.0000 | 0.0000 | 0.0000 | 0.0000 | 0.0000 | 0.0000 | 0.0000 | 0.0000 | 0.0000 | 0.0000 | 0.0000 | 0.0000 |  |
| g__Paenisporosarcina | 0.0000 | 0.0030 | 0.0000 | 0.0000 | 0.0000 | 0.0000 | 0.0000 | 0.0000 | 0.0000 | 0.0000 | 0.0000 | 0.0000 |  |
| g__Parabacteroides | 0.0500 | 0.0030 | 0.0030 | 0.0321 | 0.0350 | 0.0175 | 0.0933 | 0.0788 | 0.0671 | 0.1134 | 0.1260 | 0.0613 |  |
| g__Pedobacter | 0.0000 | 0.0000 | 0.0000 | 0.0000 | 0.0000 | 0.0000 | 0.0029 | 0.0000 | 0.0000 | 0.0000 | 0.0000 | 0.0000 |  |
| g__Phascolarctobacterium | 0.0000 | 0.0000 | 0.0000 | 0.0000 | 0.0000 | 0.0000 | 0.0000 | 0.0000 | 0.0000 | 0.0000 | 0.0000 | 0.0000 |  |
| g__Porphyromonas | 0.0010 | 0.0030 | 0.0000 | 0.0000 | 0.0039 | 0.0058 | 0.0000 | 0.0029 | 0.0029 | 0.0000 | 0.0000 | 0.0000 |  |
| g__Prevotella | 0.7000 | 0.4100 | 0.9800 | 1.5869 | 0.2645 | 0.5338 | 2.9230 | 1.6103 | 0.6039 | 0.8501 | 0.3921 | 0.7351 |  |
| g__Propionibacterium | 0.0030 | 0.0000 | 0.0000 | 0.0000 | 0.0000 | 0.0000 | 0.0000 | 0.0000 | 0.0000 | 0.0000 | 0.0000 | 0.0000 |  |
| g__Proteus | 0.0030 | 0.0100 | 0.0000 | 0.0000 | 0.0000 | 0.0000 | 0.0000 | 0.0000 | 0.0000 | 0.0100 | 0.0000 | 0.0000 |  |
| g__Pseudomonas | 0.0000 | 0.0000 | 0.0000 | 0.0000 | 0.0000 | 0.0058 | 0.0000 | 0.0000 | 0.0000 | 0.0000 | 0.0000 | 0.0000 |  |
| g__rc4-4 | 0.0030 | 0.0000 | 0.0000 | 0.0000 | 0.0000 | 0.0000 | 0.0233 | 0.0000 | 0.0000 | 0.0000 | 0.0000 | 0.0000 |  |
| g__Rhodoplanes | 0.0000 | 0.0000 | 0.0000 | 0.0000 | 0.0000 | 0.0000 | 0.0000 | 0.0000 | 0.0000 | 0.0000 | 0.0000 | 0.0000 |  |

**Supplementary Table 3 continued.** The abundance of bacteria genus in mice with different timepoint and different group in this study.

| **Genus** | **Addiction (abundance %)** | | | | | | | | | | | | |
| --- | --- | --- | --- | --- | --- | --- | --- | --- | --- | --- | --- | --- | --- |
|  | **0d** | | | **7d** | | | **30d** | | | **60d** | | | |
|  | **C** | **S** | **M** | **C** | **S** | **M** | **C** | **S** | **M** | **C** | **S** | **M** |  |
| g__Roseburia | 0.0100 | 0.5200 | 0.0700 | 0.0146 | 0.1517 | 0.0408 | 0.0554 | 0.0058 | 0.0175 | 0.0033 | 0.1680 | 0.0088 |  |
| g__Rothia | 0.0100 | 0.0030 | 0.0000 | 0.0029 | 0.0078 | 0.0204 | 0.0000 | 0.0088 | 0.0000 | 0.0000 | 0.0000 | 0.0000 |  |
| g__Ruminococcus | 0.9500 | 1.1000 | 1.1990 | 3.4685 | 0.7662 | 2.7246 | 0.8285 | 1.3769 | 0.9160 | 1.6303 | 1.6103 | 0.3676 |  |
| g__Saccharopolyspora | 0.0000 | 0.0000 | 0.0000 | 0.0000 | 0.0000 | 0.0000 | 0.0000 | 0.0029 | 0.0000 | 0.0000 | 0.0000 | 0.0000 |  |
| g__Sanguibacter | 0.0000 | 0.0000 | 0.0000 | 0.0000 | 0.0000 | 0.0000 | 0.0000 | 0.0000 | 0.0000 | 0.0000 | 0.0000 | 0.0000 |  |
| g__Schwartzia | 0.0000 | 0.0000 | 0.0000 | 0.0000 | 0.0000 | 0.0000 | 0.0000 | 0.0000 | 0.0000 | 0.0000 | 0.0000 | 0.0000 |  |
| g__Selenomonas | 0.0100 | 0.0030 | 0.0030 | 0.0000 | 0.0000 | 0.0088 | 0.0058 | 0.0058 | 0.0000 | 0.0000 | 0.0000 | 0.0000 |  |
| g__Shigella | 0.0030 | 0.0000 | 0.0000 | 0.0000 | 0.0000 | 0.0029 | 0.0000 | 0.0000 | 0.0000 | 0.0000 | 0.0000 | 0.0000 |  |
| g__SMB53 | 0.0000 | 0.0600 | 0.0000 | 0.0000 | 0.0000 | 0.0000 | 0.0000 | 0.0000 | 0.0000 | 0.0000 | 0.0000 | 0.0000 |  |
| g__Spirodela | 0.0000 | 0.0000 | 0.0000 | 0.0000 | 0.0000 | 0.0000 | 0.0000 | 0.0000 | 0.0000 | 0.0000 | 0.0000 | 0.0000 |  |
| g__Sporosarcina | 0.0000 | 0.0000 | 0.0000 | 0.0000 | 0.0000 | 0.0029 | 0.0000 | 0.0000 | 0.0000 | 0.0000 | 0.0000 | 0.0000 |  |
| g__Staphylococcus | 0.0030 | 0.0100 | 0.0000 | 0.0000 | 0.0039 | 0.0058 | 0.0058 | 0.0058 | 0.0000 | 0.0067 | 0.0000 | 0.0000 |  |
| g__Streptococcus | 0.0260 | 0.0200 | 0.0060 | 0.0117 | 0.0117 | 0.0379 | 0.0204 | 0.0204 | 0.0029 | 0.0000 | 0.0047 | 0.0000 |  |
| g__Streptomyces | 0.0000 | 0.0000 | 0.0000 | 0.0000 | 0.0000 | 0.0000 | 0.0000 | 0.0000 | 0.0000 | 0.0000 | 0.0047 | 0.0000 |  |
| g__Sutterella | 0.4800 | 0.2100 | 0.2300 | 0.4551 | 0.4201 | 0.1809 | 0.7060 | 0.6243 | 0.7730 | 1.4802 | 1.2089 | 0.6651 |  |
| g__Tannerella | 0.0000 | 0.0000 | 0.0000 | 0.0000 | 0.0000 | 0.0000 | 0.0000 | 0.0000 | 0.0000 | 0.0000 | 0.0000 | 0.0000 |  |
| g__TG5 | 0.0030 | 0.0000 | 0.0000 | 0.0000 | 0.0000 | 0.0029 | 0.0000 | 0.0000 | 0.0000 | 0.0000 | 0.0000 | 0.0000 |  |
| g__Trabulsiella | 0.0000 | 0.0030 | 0.0000 | 0.0000 | 0.0000 | 0.0029 | 0.0000 | 0.0000 | 0.0000 | 0.0000 | 0.0000 | 0.0000 |  |
| g__Treponema | 0.0000 | 0.0000 | 0.0030 | 0.0000 | 0.0000 | 0.0000 | 0.0000 | 0.0000 | 0.0000 | 0.0000 | 0.0000 | 0.0000 |  |

**Supplementary Table 3** **continued.** The abundance of bacteria genus in mice with different timepoint and different group in this study.

| **Genus** | **Addiction (abundance %)** | | | | | | | | | | | | |
| --- | --- | --- | --- | --- | --- | --- | --- | --- | --- | --- | --- | --- | --- |
|  | **0d** | | | **7d** | | | **30d** | | | **60d** | | | |
|  | **C** | **S** | **M** | **C** | **S** | **M** | **C** | **S** | **M** | **C** | **S** | **M** |  |
| g__Turicibacter | 0.0000 | 0.1100 | 0.1000 | 0.0058 | 0.0078 | 0.0817 | 0.0554 | 0.0146 | 1.7240 | 1.7003 | 3.8786 | 4.6645 |  |
| g__Vagococcus | 0.0120 | 0.0270 | 0.0000 | 0.0000 | 0.0000 | 0.0000 | 0.0000 | 0.0000 | 0.0000 | 0.0000 | 0.0000 | 0.0000 |  |
| g__Veillonella | 0.0150 | 0.0030 | 0.0000 | 0.0029 | 0.0000 | 0.0117 | 0.0000 | 0.0088 | 0.0000 | 0.0000 | 0.0000 | 0.0000 |  |
| g__Zea | 0.0000 | 0.0030 | 0.0000 | 0.0029 | 0.0000 | 0.0000 | 0.0000 | 0.0000 | 0.0000 | 0.0033 | 0.0000 | 0.0000 |  |
| g__Not assigned | 74.0900 | 78.3800 | 80.0000 | 78.4160 | 82.4193 | 86.4965 | 76.2952 | 88.0863 | 62.0012 | 83.9573 | 83.1225 | 37.9113 |  |

**Supplementary Table 3 continued.** The abundance of bacteria genus in mice with different timepoint and different group in this study.

| **Genus** | **Abstinence (abundance %)** | | | | | | | | |
| --- | --- | --- | --- | --- | --- | --- | --- | --- | --- |
|  | **1d** | | | **30d** | | | **60d** | | |
|  | **C** | **S** | **M** | **C** | **S** | **M** | **C** | **S** | **M** |
| g__[Prevotella] | 0.5688 | 0.8897 | 0.2159 | 0.6826 | 0.2367 | 0.5868 | 0.7993 | 0.3530 | 0.8489 |
| g__[Ruminococcus] | 0.2859 | 0.3763 | 0.1167 | 0.3267 | 0.3534 | 0.5334 | 0.2888 | 0.8051 | 0.1663 |
| g__02d06 | 0.0000 | 0.0000 | 0.0000 | 0.0000 | 0.0000 | 0.0000 | 0.0000 | 0.0000 | 0.0000 |
| g__Acinetobacter | 0.0000 | 0.0000 | 0.0000 | 0.0000 | 0.0000 | 0.0033 | 0.0000 | 0.0000 | 0.0000 |
| g__Actinomyces | 0.0000 | 0.0029 | 0.0000 | 0.0000 | 0.0067 | 0.0033 | 0.0000 | 0.0000 | 0.0000 |
| g__Actinopolymorpha | 0.0000 | 0.0000 | 0.0000 | 0.0000 | 0.0000 | 0.0000 | 0.0000 | 0.0000 | 0.0000 |
| g__Adlercreutzia | 0.3326 | 0.6505 | 0.2100 | 0.3151 | 0.4867 | 0.2934 | 0.1750 | 0.3588 | 0.1750 |
| g__Aggregatibacter | 0.0000 | 0.0000 | 0.0000 | 0.0000 | 0.0000 | 0.0000 | 0.0000 | 0.0000 | 0.0000 |
| g__Akkermansia | 0.0088 | 0.0438 | 20.1138 | 4.3291 | 2.3504 | 18.6864 | 9.2007 | 5.3967 | 12.3191 |
| g__Alistipes | 0.0029 | 0.0117 | 0.0058 | 0.0029 | 0.0033 | 0.0100 | 0.0088 | 0.0029 | 0.0058 |
| g__Allobaculum | 2.0391 | 0.1721 | 0.0642 | 0.3471 | 0.1000 | 0.0167 | 0.0583 | 0.1254 | 0.0554 |
| g__Anaerofustis | 0.0000 | 0.0000 | 0.0029 | 0.0000 | 0.0000 | 0.0000 | 0.0000 | 0.0000 | 0.0000 |
| g__Anaeroplasma | 0.0000 | 0.0000 | 0.0000 | 0.0000 | 0.0000 | 0.0000 | 0.0088 | 0.0000 | 0.0000 |
| g__Anaerostipes | 0.0408 | 0.0204 | 0.0000 | 0.0088 | 0.0133 | 0.0000 | 0.0233 | 0.0117 | 0.0000 |
| g__Anaerotruncus | 0.0000 | 0.0000 | 0.0000 | 0.0000 | 0.0000 | 0.0000 | 0.0000 | 0.0000 | 0.0000 |
| g__Anaerovorax | 0.0000 | 0.0000 | 0.0000 | 0.0000 | 0.0000 | 0.0000 | 0.0000 | 0.0000 | 0.0000 |
| g__Arthrobacter | 0.0000 | 0.0000 | 0.0058 | 0.0000 | 0.0000 | 0.0033 | 0.0000 | 0.0058 | 0.0000 |
| g__Atopobium | 0.0000 | 0.0000 | 0.0000 | 0.0000 | 0.0000 | 0.0000 | 0.0000 | 0.0000 | 0.0000 |
| g__Bacillus | 0.0000 | 0.0000 | 0.0000 | 0.0000 | 0.0000 | 0.0033 | 0.0000 | 0.0000 | 0.0000 |

**Supplementary Table 3 continued.** The abundance of bacteria genus in mice with different timepoint and different group in this study.

| **Genus** | **Abstinence (abundance %)** | | | | | | | | |
| --- | --- | --- | --- | --- | --- | --- | --- | --- | --- |
|  | **1d** | | | **30d** | | | **60d** | | |
|  | **C** | **S** | **M** | **C** | **S** | **M** | **C** | **S** | **M** |
| g__Bacteroides | 2.0566 | 3.3839 | 1.2660 | 1.1027 | 0.9635 | 1.4536 | 2.7509 | 1.5548 | 3.4072 |
| g__Bifidobacterium | 2.6779 | 1.8961 | 10.5951 | 3.0251 | 0.4167 | 0.3001 | 3.5706 | 0.7585 | 0.0758 |
| g__Bilophila | 0.1342 | 0.1371 | 0.0554 | 0.0963 | 0.0667 | 0.1467 | 0.1459 | 0.1663 | 0.2042 |
| g__Blautia | 0.0000 | 0.0000 | 0.0000 | 0.0000 | 0.0000 | 0.0000 | 0.0000 | 0.0000 | 0.0000 |
| g__Butyricimonas | 0.2771 | 0.2217 | 0.1254 | 0.3559 | 0.2534 | 0.2834 | 0.1809 | 0.1721 | 0.2480 |
| g__Candidatus Arthromitus | 0.0175 | 0.0146 | 0.0000 | 0.0029 | 0.0100 | 0.0200 | 0.0058 | 0.0292 | 0.0000 |
| g__Capnocytophaga | 0.0000 | 0.0000 | 0.0000 | 0.0000 | 0.0000 | 0.0000 | 0.0000 | 0.0000 | 0.0000 |
| g__Catonella | 0.0000 | 0.0029 | 0.0000 | 0.0000 | 0.0000 | 0.0000 | 0.0000 | 0.0000 | 0.0000 |
| g__Citrobacter | 0.0000 | 0.0000 | 0.0000 | 0.0000 | 0.0000 | 0.0000 | 0.0000 | 0.0000 | 0.0000 |
| g__Clostridium | 0.0029 | 0.0058 | 0.0088 | 0.0350 | 0.0000 | 0.0833 | 0.0175 | 0.0467 | 0.0029 |
| g__Cohnella | 0.0000 | 0.0000 | 0.0000 | 0.0000 | 0.0000 | 0.0000 | 0.0000 | 0.0000 | 0.0000 |
| g__Coprobacillus | 0.0700 | 0.1050 | 0.0146 | 0.0438 | 0.0700 | 0.0067 | 0.0438 | 0.0204 | 0.0496 |
| g__Coprococcus | 0.3355 | 0.3530 | 0.2713 | 0.1284 | 0.4934 | 0.2600 | 0.1021 | 0.3792 | 0.1779 |
| g__Corynebacterium | 0.0058 | 0.0000 | 0.0000 | 0.0000 | 0.0000 | 0.0000 | 0.0000 | 0.0146 | 0.0000 |
| g__Dehalobacterium | 0.1429 | 0.1021 | 0.0350 | 0.1050 | 0.1167 | 0.1367 | 0.1371 | 0.1225 | 0.0788 |
| g__Dialister | 0.0000 | 0.0000 | 0.0000 | 0.0000 | 0.0000 | 0.0000 | 0.0000 | 0.0000 | 0.0000 |
| g__Dorea | 0.1079 | 0.0875 | 0.0438 | 0.0438 | 0.0833 | 0.0733 | 0.1196 | 0.0263 | 0.0700 |
| g__Enterococcus | 0.0992 | 0.0321 | 0.0000 | 0.0438 | 0.0067 | 0.0067 | 0.0438 | 0.0175 | 0.0175 |
| g__Erwinia | 0.0000 | 0.0000 | 0.0000 | 0.0000 | 0.0000 | 0.0067 | 0.0000 | 0.0029 | 0.0000 |

**Supplementary Table 3 continued.** The abundance of bacteria genus in mice with different timepoint and different group in this study.

| **Genus** | **Abstinence (abundance %)** | | | | | | | | |
| --- | --- | --- | --- | --- | --- | --- | --- | --- | --- |
|  | **1d** | | | **30d** | | | **60d** | | |
|  | **C** | **S** | **M** | **C** | **S** | **M** | **C** | **S** | **M** |
| g__Exiguobacterium | 0.0000 | 0.0000 | 0.0000 | 0.0000 | 0.0000 | 0.0000 | 0.0000 | 0.0000 | 0.0029 |
| g__Flavisolibacter | 0.0000 | 0.0000 | 0.0000 | 0.0000 | 0.0000 | 0.0000 | 0.0000 | 0.0000 | 0.0000 |
| g__Flavobacterium | 0.0000 | 0.0000 | 0.0000 | 0.0000 | 0.0033 | 0.0000 | 0.0029 | 0.0000 | 0.0000 |
| g__Fusobacterium | 0.0029 | 0.0088 | 0.0000 | 0.0000 | 0.0000 | 0.0000 | 0.0000 | 0.0000 | 0.0000 |
| g__Gemella | 0.0000 | 0.0000 | 0.0000 | 0.0000 | 0.0000 | 0.0000 | 0.0029 | 0.0000 | 0.0000 |
| g__Geosporobacter_Thermotalea | 0.0000 | 0.0000 | 0.0000 | 0.0000 | 0.0000 | 0.0000 | 0.0000 | 0.0000 | 0.0000 |
| g__Granulicatella | 0.0058 | 0.0000 | 0.0000 | 0.0000 | 0.0000 | 0.0000 | 0.0000 | 0.0000 | 0.0000 |
| g__Haemophilus | 0.0000 | 0.0029 | 0.0000 | 0.0000 | 0.0000 | 0.0033 | 0.0000 | 0.0000 | 0.0029 |
| g__Janthinobacterium | 0.0000 | 0.0000 | 0.0000 | 0.0000 | 0.0000 | 0.0033 | 0.0000 | 0.0000 | 0.0000 |
| g__Kaistobacter | 0.0000 | 0.0000 | 0.0000 | 0.0000 | 0.0000 | 0.0033 | 0.0000 | 0.0000 | 0.0000 |
| g__Klebsiella | 0.0000 | 0.0000 | 0.0000 | 0.0000 | 0.0000 | 0.0000 | 0.0000 | 0.0000 | 0.0000 |
| g__Kocuria | 0.0029 | 0.0000 | 0.0000 | 0.0000 | 0.0000 | 0.0000 | 0.0000 | 0.0000 | 0.0000 |
| g__Lachnobacterium | 0.0000 | 0.0000 | 0.0000 | 0.0000 | 0.0000 | 0.0000 | 0.0000 | 0.0000 | 0.0000 |
| g__Lactobacillus | 6.2865 | 6.4527 | 17.8646 | 4.8658 | 2.2137 | 1.7136 | 4.6033 | 4.0519 | 4.5216 |
| g__Lactococcus | 0.0029 | 0.0000 | 0.0000 | 0.0000 | 0.0000 | 0.0000 | 0.0000 | 0.0000 | 0.0000 |
| g__Lautropia | 0.0000 | 0.0000 | 0.0000 | 0.0000 | 0.0000 | 0.0033 | 0.0000 | 0.0000 | 0.0000 |
| g__Leptotrichia | 0.0000 | 0.0000 | 0.0000 | 0.0000 | 0.0000 | 0.0000 | 0.0029 | 0.0000 | 0.0000 |
| g__Luteimonas | 0.0000 | 0.0000 | 0.0000 | 0.0000 | 0.0000 | 0.0000 | 0.0000 | 0.0000 | 0.0000 |
| g__Megasphaera | 0.0000 | 0.0000 | 0.0000 | 0.0000 | 0.0033 | 0.0000 | 0.0000 | 0.0000 | 0.0000 |

**Supplementary Table 3 continued.** The abundance of bacteria genus in mice with different timepoint and different group in this study.

| **Genus** | **Abstinence (abundance %)** | | | | | | | | |
| --- | --- | --- | --- | --- | --- | --- | --- | --- | --- |
|  | **1d** | | | **30d** | | | **60d** | | |
|  | **C** | **S** | **M** | **C** | **S** | **M** | **C** | **S** | **M** |
| g__Morganella | 0.0088 | 0.0000 | 0.0029 | 0.0000 | 0.0000 | 0.0000 | 0.0000 | 0.0000 | 0.0000 |
| g__Moryella | 0.0000 | 0.0000 | 0.0000 | 0.0000 | 0.0000 | 0.0000 | 0.0000 | 0.0000 | 0.0000 |
| g__Mucispirillum | 0.0525 | 0.0233 | 0.0233 | 0.0379 | 0.0500 | 0.1000 | 0.0817 | 0.0875 | 0.1313 |
| g__Neisseria | 0.0029 | 0.0088 | 0.0000 | 0.0000 | 0.0033 | 0.0033 | 0.0000 | 0.0058 | 0.0000 |
| g__Odoribacter | 0.4872 | 0.5659 | 0.3734 | 0.4609 | 0.4134 | 0.4834 | 0.5105 | 0.3180 | 0.6214 |
| g__Oribacterium | 0.0000 | 0.0000 | 0.0000 | 0.0000 | 0.0000 | 0.0033 | 0.0000 | 0.0000 | 0.0000 |
| g__Oscillospira | 2.3133 | 2.1762 | 0.8810 | 1.9428 | 1.1769 | 1.3636 | 1.8991 | 1.6015 | 2.0449 |
| g__Paenibacillus | 0.0029 | 0.0000 | 0.0000 | 0.0000 | 0.0000 | 0.0000 | 0.0000 | 0.0000 | 0.0000 |
| g__Paenisporosarcina | 0.0000 | 0.0000 | 0.0000 | 0.0000 | 0.0000 | 0.0000 | 0.0000 | 0.0000 | 0.0000 |
| g__Parabacteroides | 0.4551 | 0.4288 | 0.1459 | 0.2013 | 0.1134 | 0.1534 | 0.5047 | 0.1721 | 0.3996 |
| g__Pedobacter | 0.0000 | 0.0000 | 0.0000 | 0.0000 | 0.0000 | 0.0000 | 0.0000 | 0.0000 | 0.0000 |
| g__Phascolarctobacterium | 0.0029 | 0.0000 | 0.0000 | 0.0000 | 0.0000 | 0.0000 | 0.0000 | 0.0000 | 0.0000 |
| g__Porphyromonas | 0.0000 | 0.0029 | 0.0000 | 0.0000 | 0.0000 | 0.0033 | 0.0000 | 0.0000 | 0.0000 |
| g__Prevotella | 2.4971 | 3.8565 | 0.7235 | 1.2835 | 0.4567 | 0.9968 | 2.9230 | 1.6861 | 0.7118 |
| g__Propionibacterium | 0.0000 | 0.0000 | 0.0000 | 0.0000 | 0.0000 | 0.0000 | 0.0000 | 0.0000 | 0.0000 |
| g__Proteus | 0.0058 | 0.0000 | 0.0000 | 0.0058 | 0.0000 | 0.0067 | 0.0117 | 0.0000 | 0.0000 |
| g__Pseudomonas | 0.0029 | 0.0058 | 0.0000 | 0.0000 | 0.0000 | 0.0033 | 0.0000 | 0.0058 | 0.0000 |
| g__rc4-4 | 0.0000 | 0.0000 | 0.0000 | 0.0000 | 0.0000 | 0.0000 | 0.0000 | 0.0000 | 0.0000 |
| g__Rhodoplanes | 0.0000 | 0.0000 | 0.0000 | 0.0000 | 0.0000 | 0.0000 | 0.0000 | 0.0000 | 0.0029 |

**Supplementary Table 3 continued.** The abundance of bacteria genus in mice with different timepoint and different group in this study.

| **Genus** | **Abstinence (abundance %)** | | | | | | | | |
| --- | --- | --- | --- | --- | --- | --- | --- | --- | --- |
|  | **1d** | | | **30d** | | | **60d** | | |
|  | **C** | **S** | **M** | **C** | **S** | **M** | **C** | **S** | **M** |
| g__Roseburia | 0.0000 | 0.0379 | 0.0175 | 0.0292 | 0.0133 | 0.0233 | 0.0000 | 0.0000 | 0.0000 |
| g__Rothia | 0.0000 | 0.0029 | 0.0000 | 0.0000 | 0.0000 | 0.0067 | 0.0000 | 0.0058 | 0.0000 |
| g__Ruminococcus | 1.7999 | 2.2958 | 0.6534 | 1.0589 | 1.5236 | 0.7735 | 1.3623 | 1.1756 | 0.9160 |
| g__Saccharopolyspora | 0.0000 | 0.0000 | 0.0000 | 0.0000 | 0.0000 | 0.0000 | 0.0000 | 0.0000 | 0.0000 |
| g__Sanguibacter | 0.0000 | 0.0000 | 0.0000 | 0.0029 | 0.0000 | 0.0000 | 0.0000 | 0.0000 | 0.0000 |
| g__Schwartzia | 0.0000 | 0.0000 | 0.0000 | 0.0000 | 0.0000 | 0.0000 | 0.0000 | 0.0029 | 0.0000 |
| g__Selenomonas | 0.0000 | 0.0000 | 0.0000 | 0.0000 | 0.0000 | 0.0033 | 0.0029 | 0.0029 | 0.0000 |
| g__Shigella | 0.0000 | 0.0000 | 0.0000 | 0.0000 | 0.0000 | 0.0000 | 0.0000 | 0.0000 | 0.0000 |
| g__SMB53 | 0.0000 | 0.0000 | 0.0000 | 0.0000 | 0.0000 | 0.0000 | 0.0175 | 0.0088 | 0.0000 |
| g__Spirodela | 0.0029 | 0.0000 | 0.0000 | 0.0000 | 0.0000 | 0.0000 | 0.0029 | 0.0000 | 0.0000 |
| g__Sporosarcina | 0.0000 | 0.0000 | 0.0088 | 0.0000 | 0.0000 | 0.0000 | 0.0000 | 0.0146 | 0.0029 |
| g__Staphylococcus | 0.0000 | 0.0175 | 0.0000 | 0.0029 | 0.0133 | 0.0000 | 0.0117 | 0.0058 | 0.0000 |
| g__Streptococcus | 0.0146 | 0.0204 | 0.0000 | 0.0029 | 0.0333 | 0.0100 | 0.0058 | 0.0088 | 0.0000 |
| g__Streptomyces | 0.0000 | 0.0000 | 0.0000 | 0.0000 | 0.0000 | 0.0000 | 0.0000 | 0.0000 | 0.0000 |
| g__Sutterella | 1.2194 | 0.8985 | 1.0852 | 0.6768 | 0.5334 | 0.6168 | 1.2398 | 0.3238 | 0.5251 |
| g__Tannerella | 0.0000 | 0.0029 | 0.0000 | 0.0000 | 0.0000 | 0.0000 | 0.0000 | 0.0000 | 0.0000 |
| g__TG5 | 0.0000 | 0.0000 | 0.0000 | 0.0000 | 0.0000 | 0.0000 | 0.0000 | 0.0000 | 0.0000 |
| g__Trabulsiella | 0.0000 | 0.0000 | 0.0000 | 0.0000 | 0.0000 | 0.0000 | 0.0000 | 0.0000 | 0.0000 |
| g__Treponema | 0.0000 | 0.0000 | 0.0000 | 0.0000 | 0.0000 | 0.0033 | 0.0000 | 0.0000 | 0.0000 |

**Supplementary Table 3 continued.** The abundance of bacteria genus in mice with different timepoint and different group in this study.

| **Genus** | **Abstinence (abundance %)** | | | | | | | | |
| --- | --- | --- | --- | --- | --- | --- | --- | --- | --- |
|  | **1d** | | | **30d** | | | **60d** | | |
|  | **C** | **S** | **M** | **C** | **S** | **M** | **C** | **S** | **M** |
| g__Turicibacter | 2.5758 | 3.8565 | 5.7410 | 4.0432 | 2.9538 | 2.5704 | 5.0758 | 1.5782 | 0.1663 |
| g__Vagococcus | 0.0000 | 0.0000 | 0.0000 | 0.0000 | 0.0000 | 0.0000 | 0.0000 | 0.0000 | 0.0000 |
| g__Veillonella | 0.0029 | 0.0000 | 0.0000 | 0.0000 | 0.0033 | 0.0000 | 0.0029 | 0.0029 | 0.0000 |
| g__Zea | 0.0000 | 0.0000 | 0.0000 | 0.0000 | 0.0000 | 0.0067 | 0.0000 | 0.0000 | 0.0000 |
| g__Not assigned | 73.0455 | 70.8256 | 39.3291 | 74.3903 | 84.4607 | 68.2247 | 64.0548 | 78.5706 | 72.0478 |

**Supplementary Table 4.** Performance of random forest regression models for predicting time points during METH addiction.

|  | training set | testing set |
| --- | --- | --- |
| Mean absolute error | 8.091057 | 9.264646 |
| Root Mean Square Error | 9.304277 | 10.40371 |
| R^2^（%） | 80.42 | 78.18 |

**Supplementary Table 5.** Performance of random forest regression models for predicting time points during METH Abstinence.

|  | training set | testing set |
| --- | --- | --- |
| Mean absolute error | 11.344873 | 13.33853 |
| Root Mean Square Error | 12.943752 | 15.73821 |
| R^2^（%） | 48.08 | 45.92 |
